# Fluorinated terpenoids and their fluorine-containing derivatives

**DOI:** 10.1039/d5ra09047d

**Published:** 2026-02-04

**Authors:** Muhammad Badrul Huda, Nurlelasari, Unang Supratman, Yudha P. Budiman

**Affiliations:** a Department of Chemistry, Faculty of Mathematics and Natural Sciences, Universitas Padjadjaran Jl. Raya Bandung-Sumedang Km 21 Sumedang 45363 West Java Indonesia y.p.budiman@unpad.ac.id; b Department of Chemistry, Faculty of Sciences and Mathematics, Universitas Diponegoro Jl. Prof. Soedarto No. 13 Tembalang Semarang 50275 Central Java Indonesia; c Central Laboratory, Universitas Padjadjaran Jl. Raya Bandung-Sumedang Km 21 Sumedang 45363 West Java Indonesia

## Abstract

Fluorine-containing molecules have attracted increasing attention in pharmaceutical chemistry owing to their unique physicochemical properties. As there are no known examples of naturally occurring fluorinated organic molecules, fluorination of organic molecules gains significant interest in organic synthesis. Terpenoids represent one of the largest and most structurally diverse classes of natural products, exhibiting a broad range of pharmacological effects. Fluorination of terpenoid scaffolds has therefore become an active area of research aimed at enhancing or diversifying their bioactivity profiles. This review summarizes the synthetic strategies developed for the preparation of fluorinated terpenoids across different subclasses, including mono-, sesqui-, di-, tri-, and tetranor-terpenes.

## Abstract 2

Synthetic methods for the fluorination of terpenoids have enabled the development of fluorinated terpenoids as important pharmaceuticals.

## Graphical abstract

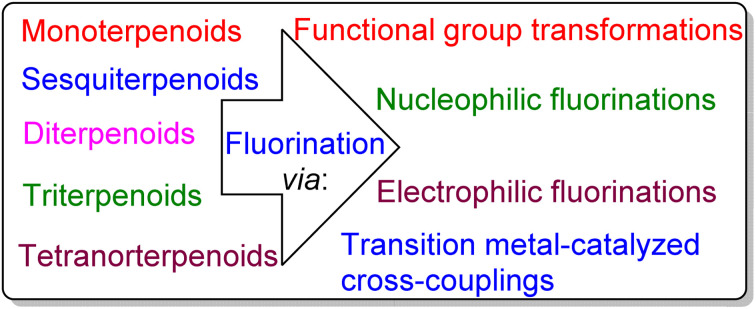

## 1. Introduction

Terpenoids represent the most structurally diverse class of natural products, comprising more than 80 000 known compounds distributed across all kingdoms of life.^1^ Naturally occurring terpenoids exhibit a remarkably broad spectrum of biological activities, including anticancer,^2^ anti-inflammatory,^3^ antimalarial,^4^ cytotoxic,^5^ antiviral, as well as hepato- and cardioprotective properties.^6^ In parallel, the incorporation of fluorine into organic molecules has gained significant attention, particularly in pharmaceutical and agrochemical synthesis, owing to its profound impact on metabolic stability, bioavailability, and overall biological performance.^7–14^

In recent years, approximately 25% of all marketed drugs have been fluoro-pharmaceuticals, representing a remarkably high proportion compared to other halogen-containing therapeutics, despite the natural abundance of organochlorine and organobromine compounds.^15^ Notably, several clinically relevant fluorinated pharmaceuticals belong to the terpenoid family and exhibit potent anticancer and anti-inflammatory activities. The unique properties of the fluorine atom—its high electronegativity, small atomic radius, and the low polarizability of the C–F bond—can profoundly influence the behaviour of a molecule within biological systems.^16–18^ More broadly, the introduction of fluorine can modulate key pharmacological processes, including receptor or enzyme binding, metabolic stability and clearance, absorption and transport, and the modulation or inhibition of enzymatic pathways.^19–22^

**Fig. 1:**
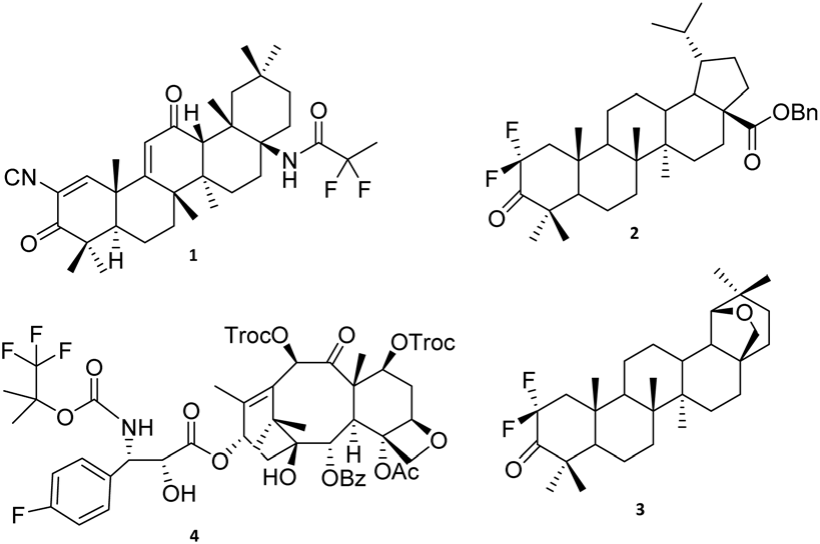
Representative fluorinated terpenoids and their significance as pharmaceutical agents. (1) Omaveloxolone, a therapeutic agent for neurodegenerative diseases.^1^ (2) 2,2-Difluorodihydrobetulonic acid, an anticancer compound.^24^ (3) Difluoroallobetulone, an anticancer compound.^2^ (4) *N*-De-*t*-butoxycarbonyl-*N*-[2-(1,1,1-trifluoro-2-methyl)pro-pyloxycarbonyl]-30-dephenyl-30-(4-fluorophenyl)docetaxel, an antitumor agent.^3^

Several studies have demonstrated that fluorinated terpenoid derivatives often exhibit enhanced biological activity. For instance, omaveloxolone (1), a fluorinated oleanane derivative, has been approved for the treatment of Friedreich's ataxia (Fig. 1).^23^ Cytotoxicity studies have further shown that introducing a fluorine atom at the C-2 position of lupane scaffolds (*e.g.*, compounds 2 and 3) significantly improves their antitumor potency.^24^ Likewise, fluorinated docetaxel (4) displays superior anticancer activity compared with the parent drug, particularly in the treatment of advanced ovarian and breast cancers.^25^ These examples highlight the broader trend that fluorinated terpenoids represent a valuable class of bioactive molecules with considerable therapeutic potential.

The introduction of a fluorine atom in place of a hydrogen or hydroxyl group generally does not result in substantial steric perturbation. However, intramolecular interactions involving the fluorine substituent can induce notable conformational changes within the molecule. Fluorination often slows down metabolic degradation relative to the non-fluorinated parent structure. Moreover, fluorinated compounds frequently display increased chemical stability, improved solubility, and enhanced bioavailability—attributes that are partly associated with their typically higher lipophilicity.^26–29^ Despite the numerous reports describing the synthesis of various fluorinated terpenoid derivatives (including mono-, di-, sesqui-, tri-, and tetranor-terpenoids), a systematic overview of the synthetic methodologies remains lacking. This review covers reports on fluorinated terpenoids and their fluorine-containing derivatives published up to the end of 2024. In this review, we aim to fill this gap by summarizing the principal strategies for the synthesis of fluorinated terpenoids, with a particular focus on the reagents and catalysts employed.

## 2. Synthesis of fluorinated terpenoids

### 2.1. Fluorinated monoterpenoids

Natural compounds, such as the monoterpenoids found in natural essential oils, are crucial sources of numerous biological activities. Research on fluorinated monoterpenoids has been conducted by numerous researchers to increase their bioactivity. Numerous of the resulting fluorinated monoterpenoids have strong biological activity; these include cytotoxic, analgesic, pesticides, and antiviral properties.^30^

#### 2.1.1. Esterification

**Scheme 1:**
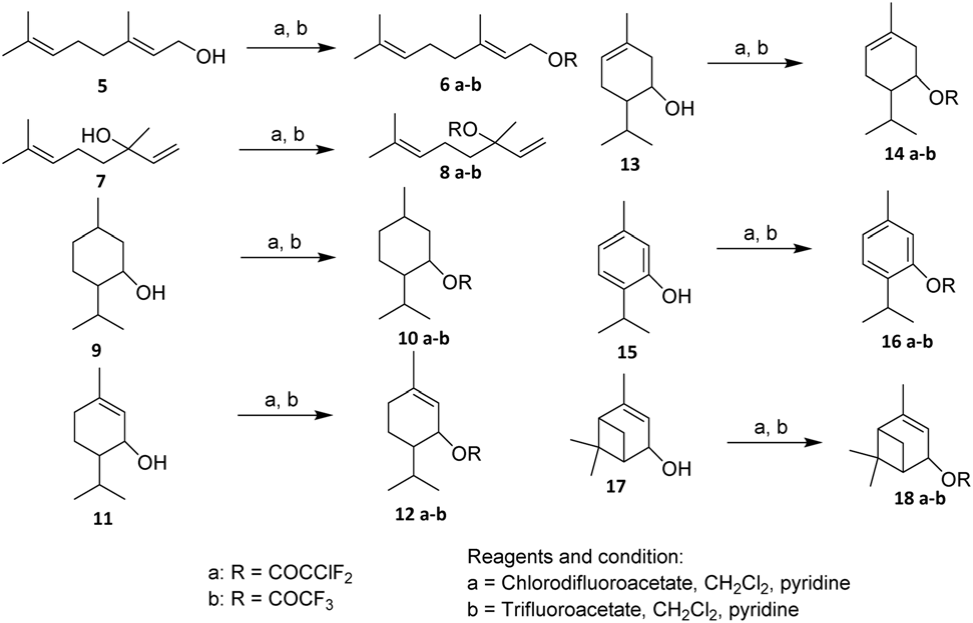
Fluorination of hydroxyl-containing compounds using fluoroalkylation esterification.^32^

Monoterpenoids are characteristically lipophilic. Therefore, they possess high potential for interference with the biochemical and physiological functions of insect herbivores.^31^ In 1994, Rice and Coats have been fluorinated some monoterpenes. Some monoterpenes are *e.g.*, Geranyl (5), Linalyl (7), Menthyl (9), 4-Carvomenthenyl (11), (–)Carvyl (13), Thymyl (15), Verbenyl (17), have been fluorinated using chlorodifluoroacetate and trifluoroacetate in methylene chloride (Scheme 1).^32^

The key transformation—esterification of the monoterpenoid alcohol—proceeded efficiently without reported side reactions such as olefin fluorination, rearrangement, or elimination, suggesting good functional group tolerance toward isolated C=C double bonds and allylic systems. This is particularly noteworthy for substrates like geraniol and linalool derivatives, where competing reactions at allylic positions could be anticipated under more aggressive fluorination conditions. The mildness of the esterification protocol thus represents a practical advantage for late-stage functionalization of terpene scaffolds.^32^ The insecticidal activities of the fourteen fluorinated monoterpen were evaluated against the larvae of *Musca domestica*. It has been found that the study revealed that the fluoroacetate derivatives of cyclic monoterpenoids exhibited the highest potency and were the most effective house fly fumigants.^32^

#### 2.1.2. Prins reactions

The Prins reaction can be applied to introduce fluorine substituents into monoterpenoid frameworks. Ilyina *et al.* reported the fluorination of monoterpenoids *via* Prins-type transformations. A representative example is the synthesis of 4-fluorohexahydro-2H-chromene derivatives (21a–h and 25), which were prepared from the widely available monoterpenoids isopulegol (19) and (−)-verbenone (22).^30,33,34^

**Scheme 2:**
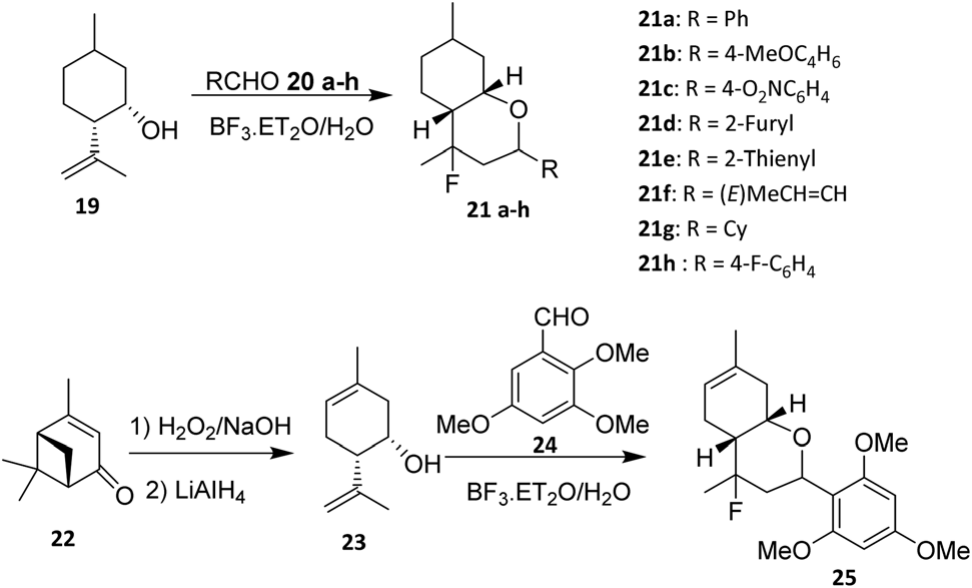
Fluorination *via* the Prins reaction.^30^

Halogenated tetrahydropyranyl rings are typically generated through Prins reactions between homoallylic alcohols and aldehydes, catalysed by suitable halogen-containing Lewis acids or ionic liquids (Scheme 2). Only a few examples describing the introduction of fluorine *via* halo-Prins cyclization have been reported to date.^35,36^ In these reactions, BF_3_·Et_2_O functions dually as the catalyst and the fluorine source.

**Scheme 3:**
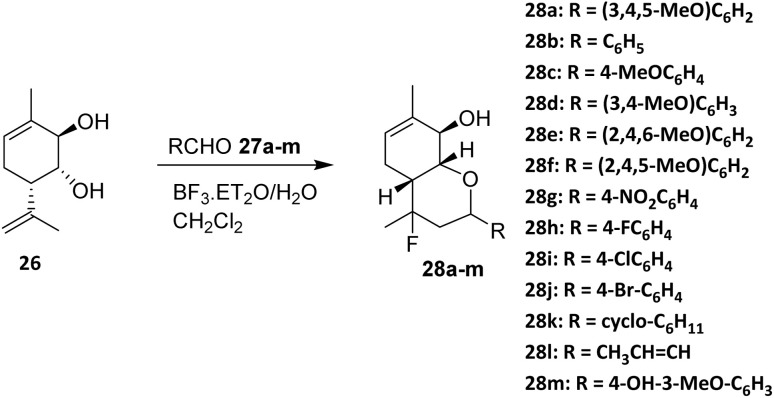
Fluorination of *para*-mentha-6,8-dien-2,3-diol (26) *via* the Prins reaction.^37^

In 2016, Michalchenko *et. al.* reported the fluorination of *para*-mentha 6,8-dien-2,3diol (26) using boron trifluoride diethyl etherate (BF_3_·Et_2_O) as the fluorine source. These research was developed to study the effect of substituents at the aldehyde aromatic ring (27a–m) on the reaction yield (Scheme 3). Among the aldehyde examined, 3,4,5-trimethoxy benzaldehyde (28a) was formed with the highest yield.^37^ The reaction tolerates terpene-derived olefins and cyclic backbones, indicating reasonable functional group compatibility within rigid monoterpenoid scaffolds.

A key determinant of reaction efficiency is the nature of the aldehyde component. Michalchenko *et al.* systematically evaluated a range of aromatic aldehydes bearing different substituents and demonstrated that electron-donating groups markedly enhance reaction yields. Notably, 3,4,5-trimethoxybenzaldehyde afforded the highest yield, which is consistent with enhanced stabilization of the oxocarbenium intermediate during the cyclization step. These results underscore the dominant role of electronic effects in governing reaction outcomes, while steric congestion on the aromatic ring can impose additional constraints.

Despite its synthetic utility, the halo-Prins fluorination strategy suffers from inherent limitations. The requirement for strong Lewis acidic conditions restricts compatibility with acid-sensitive functional groups and may limit broader application in complex, multifunctional monoterpenoid systems.

#### 2.1.3. Catalyst montmorillonite clay K10

**Scheme 4:**
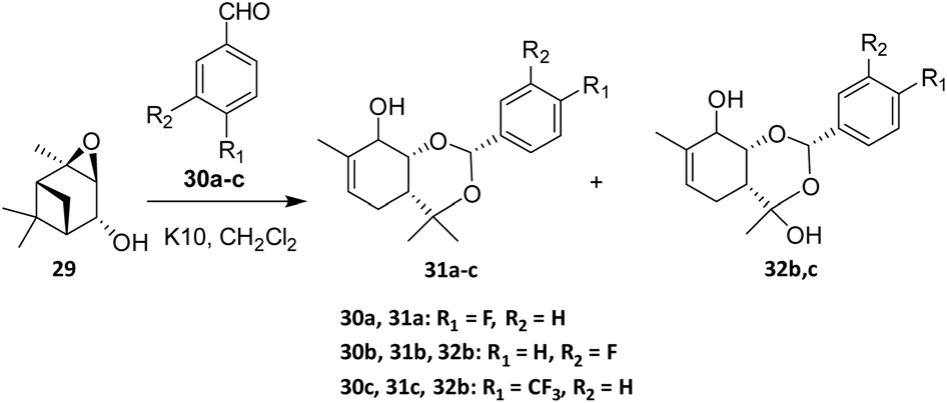
K10-clay catalyzed Prins cyclization reaction.^39^

To access new terpenoid-based compounds, Ilyina *et al.* (2010) carried out the reaction of verbenol epoxide (29) with aromatic aldehydes (30a–c) in the presence of montmorillonite clay K10 (Scheme 4). The major intermolecular products obtained from the reaction of verbenol epoxide 29 with *para*-fluorinated aromatic aldehydes 30a–c were benzodioxin derivatives 30a–c.^38^ Kurbakova *et al.* subsequently reported a related fluorination reaction of verbenol epoxide with benzaldehydes 30b and 30c, yielding compounds 32b,c.^39^ Reactions with *para*-fluorinated aromatic aldehydes predominantly afforded benzodioxin derivatives, indicating that the presence of an appropriately activated electrophile biases the reaction toward intermolecular cyclization rather than competitive rearrangement pathways.

Among the aldehydes examined, the reaction employing 4-(trifluoromethyl)benzaldehyde (30c) afforded the highest yield of intermolecular products. These observations highlight the role of electronic effects in steering reaction pathways and product distributions, providing insight into structure–reactivity relationships within this system. The selection of fluorine-containing substituents in the aldehydes was motivated by the need to investigate structure–activity relationships, particularly the influence of substituent electronic effects on the structures of the products formed in reactions with verbenol epoxide (29).^39^

#### 2.1.4. Catalyst zeolite H-Beta (KAl(SiO_4_)_2_ × 12H_2_O)

**Scheme 5:**
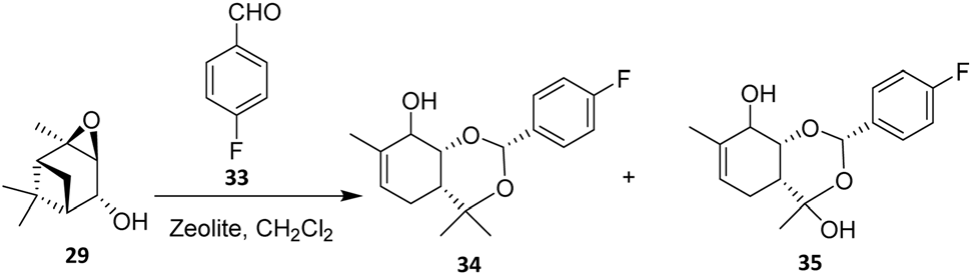
Zeolite-catalyzed Prins cyclization reaction.^40^

In 2015, Tozorova *et al.* reported the fluorination of a dioxcinol monoterpenoid derivative with potential analgesic activity, obtained from the reaction between verbenol oxide (29) and 4-fluorobenzaldehyde (33) under a heterogeneous catalytic system (Scheme 5).^40^ Zeolite H-Beta catalysts with three different SiO_2_/Al_2_O_3_ ratios (25, 150, and 300), as well as a bifunctional Fe–H-Beta-150 catalyst, were examined. This design underscores the critical role of catalyst tuning in modulating reactivity within terpene-based epoxide–aldehyde systems. As expected from pyridine desorption experiments, the concentration of acid sites increased with decreasing SiO_2_/Al_2_O_3_ ratio. The Brønsted acidity of Fe–Beta-150 was comparable to that of H-Beta-150, while the Lewis acidity increased upon introduction of iron.

The reaction between verbenol oxide 29 and 4-fluorobenzaldehyde 33 was investigated over a temperature range of 25–70 °C using either H-Beta zeolite or Fe-modified Beta catalysts. Complete conversion of verbenol oxide was achieved over H-Beta-25 in toluene using a 1 : 5 ratio of reactants, affording the isomerization products 34 and 35 as the major products. This outcome indicates that excessive acidity favors skeletal rearrangement of the terpene framework over intermolecular C–C bond formation, thereby limiting selectivity toward fluorinated products. In addition to H-Beta-25, Fe–H-Beta-150 also proved to be a promising catalyst for this transformation, yielding approximately 10% of the targeted fluorinated product under the optimized conditions.^40^ Overall, the study demonstrates that heterogeneous zeolite-based fluorination of monoterpenoids is feasible but highly sensitive to acid strength and catalyst composition, necessitating further optimization to expand substrate scope and improve selectivity.

#### 2.1.5. Click reaction (Cu catalsyt)

**Scheme 6:**
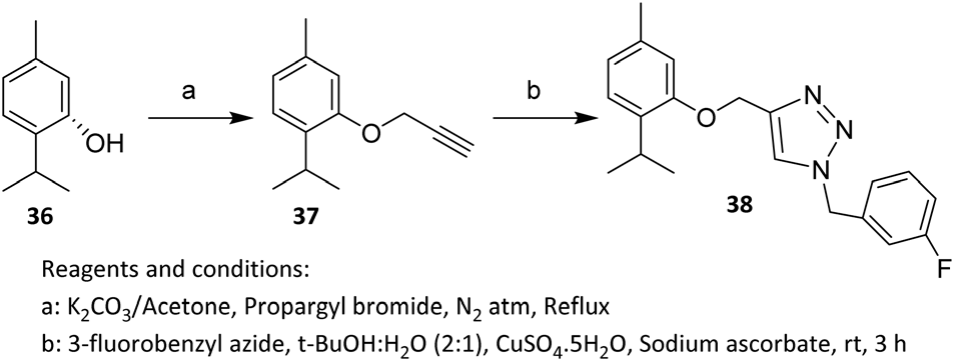
Fluorination *via* Cu(i)-catalyzed click chemistry.^42^

In 2022, Addo *et al.* reported the synthesis of fluorinated thymol derivative 38 from the alkyne precursor 37. The *O*-alkylation of thymol (36) was efficiently performed using K_2_CO_3_ and propargyl chloride in acetone at low temperature, affording the terminal alkyne intermediate *O*-propargyl thymol.^41^ Subsequently, the *O*-propargylated thymol derivative was coupled with 3-fluorobenzyl azide *via* a copper-catalysed click reaction to give the desired triazole product in a high yield of 77% (Scheme 6).^42^ The target compound was thus obtained through a standard Cu-catalysed azide–alkyne cycloaddition (CuAAC). The high yield of the CuAAC reaction is attributed to the selective activation of the terminal alkyne *via* copper(i) acetylide formation, which undergoes a highly regioselective cycloaddition with the azide to form 38. The chemical inertness of the ether-linked thymol scaffold and the stability of the fluorinated benzyl azide under the reaction conditions suppress side reactions, resulting in an efficient and clean transformation.

#### 2.1.6. Acetyltrimethylsilane and TFMTMS

**Scheme 7:**
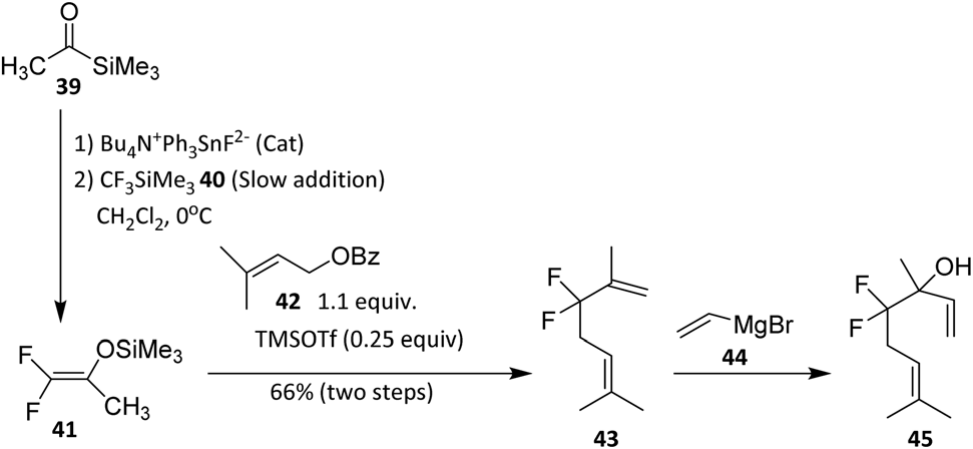
Fluoroallylation reaction to form the monoterpene 4,4-difluorolinalool.^43^

Acetyltrimethylsilane (39) and trifluoromethyltrimethylsilane (TFMTMS, 40) have proven to be valuable reagents for the synthesis of *gem*-difluoro analogues of monoterpenes. In a reported strategy,^43^ demonstrated with linalool and geraniol, the approach involves a three-component, one-pot reaction sequence followed by a simple subsequent step in the case of linalool (Scheme 7).

The key intermediate in this methodology is 1,1-difluoro-2-trimethylsilyloxypropene (41), which, upon coupling with prenyl benzoate (42), affords the difluoroheptenone derivative 43. This intermediate readily participates in coupling with prenyl benzoate to afford the difluoroheptenone derivative, indicating good compatibility with allylic esters and conjugated systems commonly found in monoterpenoids. The reaction sequence tolerates unsaturation and secondary alcohol precursors, underscoring its applicability to acyclic terpene substrates. Treatment of 43 with vinylmagnesium bromide (44) then yields the targeted *gem*-difluorinated monoterpenoid, 4,4-difluorolinalool (45).^43^ This step exploits the electrophilicity of the carbonyl group while preserving the *gem*-difluoro unit, highlighting the stabilizing effect of fluorine on adjacent carbocationic or anionic intermediates. The successful Grignard addition also indicates that the CF_2_ group does not impede nucleophilic attack, an important consideration for downstream functionalization.

#### 2.1.7. The Ruppert–Prakash reagent (TMSCF_3_)

To refine methods for the trifluoromethylation of carbonyl compounds, the trifluoromethyl group was introduced directly into the monoterpenoid skeleton using myrtenal (46). In 2021, Ilchenko *et al.* reported the nucleophilic addition of the Ruppert–Prakash reagent, (trifluoromethyl)trimethylsilane (TMSCF_3_), initiated by tetra-*n*-butylammonium fluoride trihydrate (TBAF·3H_2_O), to the carbonyl group of myrtenal 46 in THF at 0 °C under an argon atmosphere.^44^

In the first step of the reaction, the corresponding trimethylsilyl ethers 47 were formed. Subsequent acidic hydrolysis yielded the trifluoromethylated products—myrtenol derivatives 48—in a total isolated yield of 81% with a diastereomeric excess (de) of 17%. The diastereomeric excess was determined from the ratio of signal intensities of compounds 48 and 49 in the ^1^H NMR spectrum.

**Scheme 8:**
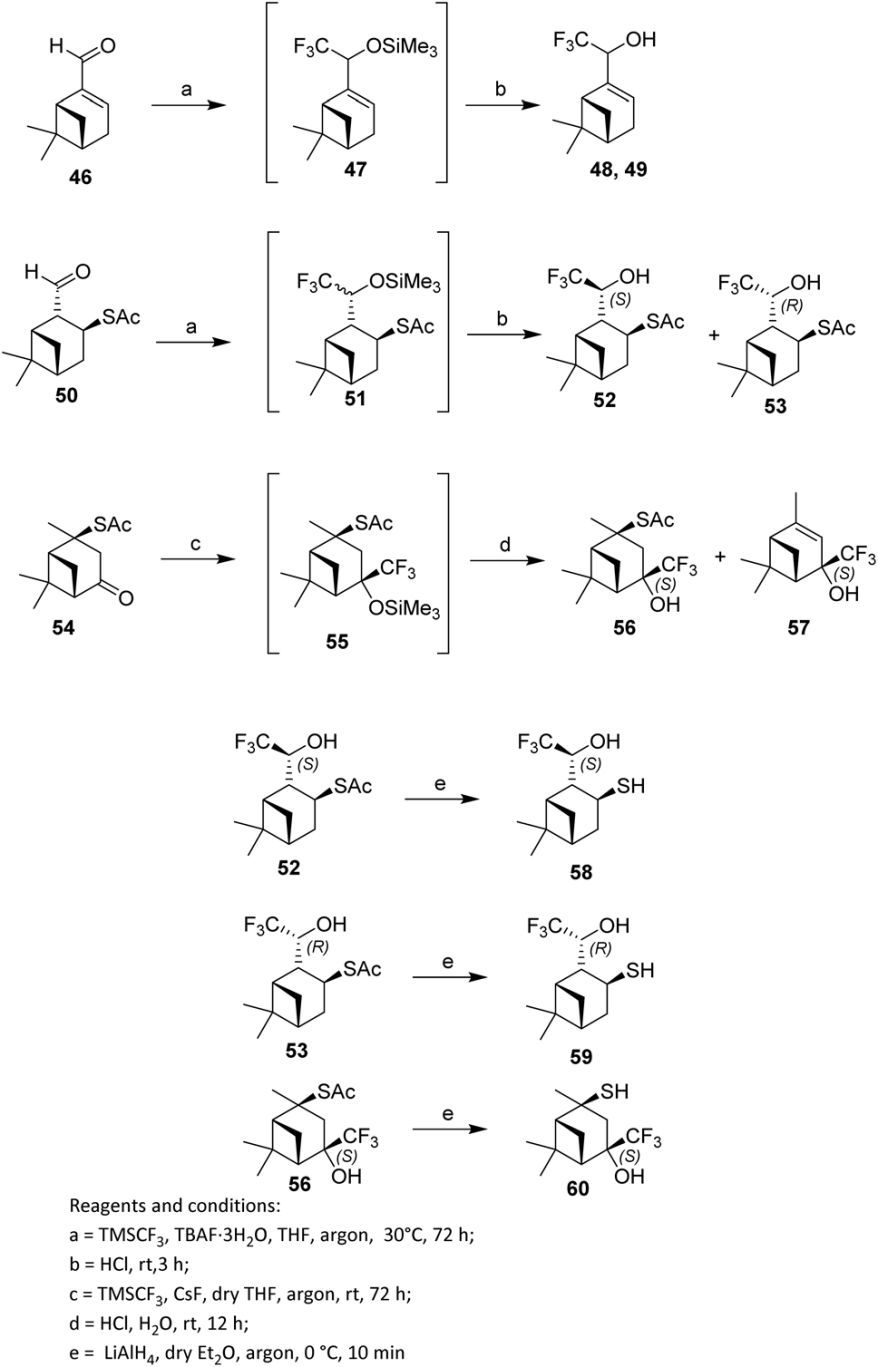
Fluorination involving the Ruppert–Prakash reagent.^44^

To avoid resinification and because the TMSCF3–TBAF·3H_2_O system is unstable under prolonged reaction times or elevated temperatures, the trifluoromethylation of 2-formylisopinocampheyl-3-thioacetate 50 was conducted at 30 °C with the reaction time extended to three days. Under these conditions, diastereomers 52 and 53 were obtained in a 52% combined yield with 42% de, with thioacetate 52 being the predominant component (Scheme 8). In contrast, thioacetate 54 did not react with the Ruppert–Prakash reagent (TMSCF3–TBAF·3H_2_O) under the same conditions, likely due to steric hindrance arising from the bulky TBAF species. Consequently, an alternative fluoride initiator, CsF, which is also employed in tandem nucleophilic CF_3_-addition protocols, was evaluated. Using the TMSCF3–CsF system, trifluoromethylated thioacetate 56 was formed as the sole (4*S*)-diastereomer in 37% yield. The reaction also produced the desulfurized product 57 in 31% yield (Scheme 8).

The deacylation of thioacetates 52, 53, and 56 with lithium aluminium hydride (LiAlH_4_) in dry Et_2_O under an argon atmosphere afforded the corresponding thiols 58, 59, and 60 in 73–90% yields, while retaining the stereochemical configuration at the stereocentres (Scheme 8).

**Scheme 9:**
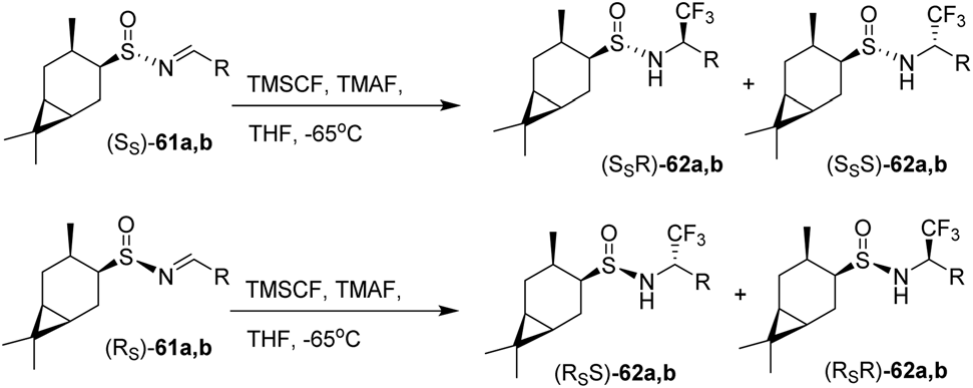
Fluorination *via* the Ruppert–Prakash reagent activated by TMAF.^45^

The trifluoromethylation of the separated diastereomers of sulfinimines 61a,b was achieved *via* nucleophilic addition of the Ruppert–Prakash reagent, TMSCF_3_ (trifluoromethyltrimethylsilane), to the C=N double bond in the presence of TMAF (tetramethylammonium fluoride) as the fluoride source in THF at −65 °C. This transformation yielded the diastereomeric sulfinamides 62a,b in 42–58% yields with 87–99% de (Scheme 9). In the study by Sudarikov *et al.* (2018), it was shown that trifluoromethylation of (SS)-*tert*-butanesulfinimines using the Ruppert–Prakash system (TMSCF_3_–TBAT/TMAF) predominantly generates the corresponding (SSR)-trifluoromethylated derivatives, while (RS)-*tert*-butanesulfinimines mainly afford the (RSS)-diastereomers. These observations are consistent with single-crystal X-ray diffraction data of sulfinamide (SSR)-62b. Combined with NMR spectroscopic data—specifically, the chemical shifts and spin–spin coupling constants of the C(1′)H and NH protons—it was concluded that the other possible diastereomers, namely (SSR)-62a, (RSS)-62a,b, (SSS)-62a,b, and (RSR)-62a,b, are formed only in trace amounts.^45^

**Scheme 10:**
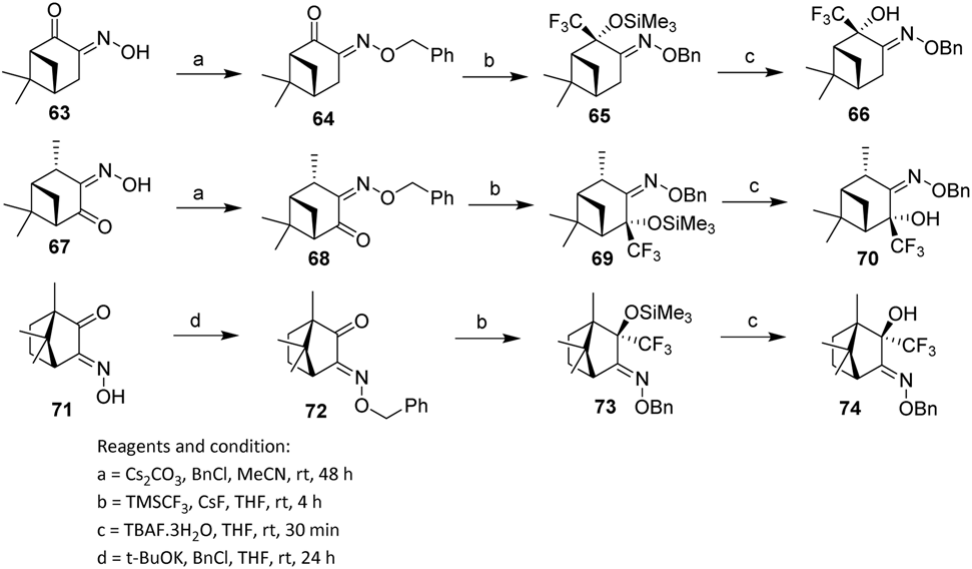
Trifluoromethylation of camphor-derived imines using the Ruppert–Prakash reagent.^46^

Petrova *et al.* employed ketooximes 63, 67, and 71, derived from (+)-nopinone, (−)-verbanone, and (+)-camphorquinone, respectively, to prepare the corresponding benzyl-*O*-oximes 64, 68, and 72. These compounds were obtained in 68%, 55%, and 45% yields, respectively. Benzyl-*O*-oximes 64, 68, and 72 possess two potential reactive centres—the C=O and C=N bonds—both of which, in principle, may undergo trifluoromethylation. As illustrated in Scheme 10, imines and sulfinimines typically react at the C=N bond, whereas monoterpene-derived ketooximes 64, 68, and 72 undergo trifluoromethylation exclusively at the C=O bond.

Nucleophilic addition of the Ruppert–Prakash reagent (trifluoromethyltrimethylsilane, TMSCF_3_) to β-keto benzyl-*O*-oximes 64, 68, and 72 at the C=O double bond was performed in THF at 4 °C under an argon atmosphere using cesium fluoride (CsF) as the initiator. In the first step, the corresponding trimethylsilyl ethers 65, 69, and 73 were formed. Subsequent treatment with tetrabutylammonium fluoride hydrate (TBAF·3H_2_O) afforded the trifluoromethylated alcohols 66, 70, and 74 in 81%, 89%, and 95% yields, respectively (Scheme 10).^46^

#### 2.1.8. Electrophilic fluorination

Ketones can serve as directing groups for aliphatic fluorination using Selectfluor in the presence of catalytic benzil and visible light. In 2017, Bume *et al.* demonstrated selective β-fluorination of rigid menthone 75, which contains two tertiary carbon centres. A visible-light-sensitized, ketone-directed C–H fluorination protocol was employed using benzil (10 mol%) as the photocatalyst, Selectfluor as the putative atomic-fluorine source,^47^ and cool white LEDs as the irradiation source.^48^ Under these mild conditions, predictable β- or γ-fluorination can be achieved depending on the spatial proximity of the target C–H bond to the carbonyl group.

**Scheme 11:**
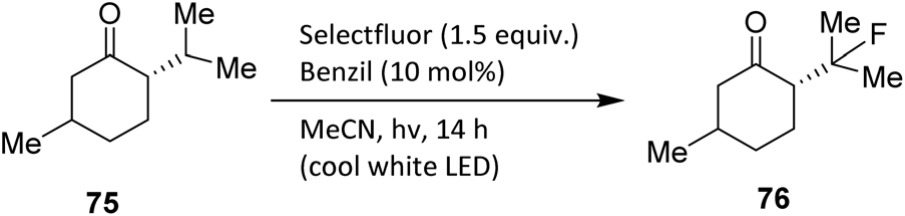
Ketone-directed C–H fluorination of menthone using Selectfluor under benzil-sensitized visible-light photocatalysis.^49^

Application of β-fluorination conditions furnished compound 76 in 55% yield, supporting the proposed involvement of the ketone functionality in directing hydrogen-atom abstraction and subsequent fluorination.^49^ Notably, alternative N–F reagents—such as *N*-fluorobenzenesulfonimide (NFSI) and *N*-fluoropyridinium tetrafluoroborate—failed to produce the fluorinated product 76 (Scheme 11). Although NFSI can react with alkyl radicals, Selectfluor reacts substantially faster and is more likely to participate in ketone-mediated electron-transfer pathways crucial for this transformation.

**Scheme 12:**
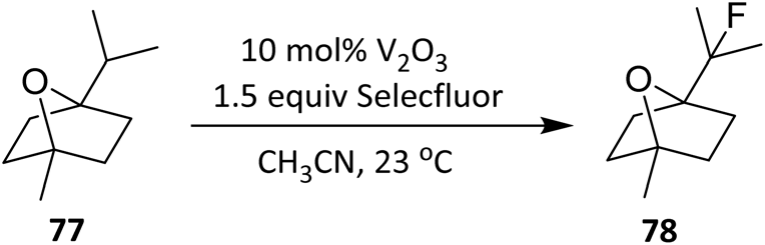
V_2_O_3_-catalyzed direct C–H fluorination of 1,4-cineole using Selectfluor.^50^

In another study, Xia *et al.* (2014) demonstrated that vanadium(iii) oxide efficiently catalyses the direct fluorination of C(sp^3^)–H bonds using Selectfluor. This protocol was applied to the monoterpene 1,4-cineole 77, affording a highly selective transformation under operationally simple conditions (Scheme 12). The heterogeneous catalyst, along with reaction by-products, can be removed readily by filtration. Fluorination occurs preferentially at the tertiary C–H site, yielding product 78 in 53% isolated yield.^50^

Fluorination occurs preferentially at the tertiary C–H position, which is both sterically accessible and energetically favored owing to its lower bond dissociation energy relative to secondary or primary C–H bonds. This regioselectivity is consistent with a radical-type mechanism, in which hydrogen atom abstraction constitutes the rate-determining step and is governed primarily by C–H bond strength rather than by electronic directing effects.

#### 2.1.9. Palladium catalyst

Palladium catalysts can mediate fluorination reactions using fluoride salts; however, controlling regioselectivity remains one of the major challenges in developing efficient Pd-catalyzed fluorination methods.^51^

**Scheme 13:**
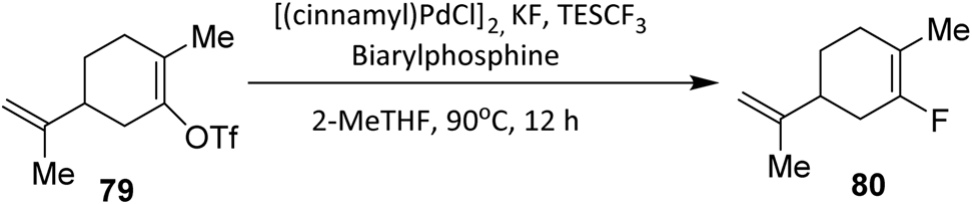
Palladium-catalyzed fluorination of monoterpenoid derivative 79 using TESCF_3_ as an additive.^51^

In 2016, Ye *et al.* reported a palladium-catalyzed fluorination of the monoterpenoid derivative 79.^52^ The key to this transformation was the addition of triethyl(trifluoromethyl)silane (TESCF_3_), which effectively addressed the regioselectivity issues commonly encountered in Pd-catalyzed fluorination. Together with a newly designed biarylphosphine ligand, TESCF_3_ enabled the development of a highly regioselective and efficient fluorination protocol. Under the optimized conditions, the desired fluorinated monoterpenoid 80 was obtained in 69% yield with excellent regioselectivity (Scheme 13).

#### 2.1.10. Comparative summary of fluorination methods for monoterpenoids

Fluorination strategies applied to monoterpenoids encompass a broad spectrum of reactivity, ranging from functional group derivatization to direct C–H fluorination, each associated with distinct advantages and limitations. Esterification-based fluoroalkylation represents one of the most straightforward and broadly applicable approaches, offering excellent functional group tolerance and compatibility with unsaturated terpene frameworks under mild conditions. However, fluorine is introduced indirectly, leaving the carbon skeleton unaltered and thus exerting only a limited impact on the electronic and steric properties of the monoterpenoid core.

In contrast, Prins-type fluorination and clay- or zeolite-catalyzed cyclizations enable skeletal reorganization and the incorporation of fluorinated aromatic units, albeit at the cost of requiring strongly Lewis acidic conditions. Consequently, their substrate scope is restricted by susceptibility to acid-promoted rearrangements, and reaction outcomes are highly sensitive to both the electronic nature of the aldehyde and the acidity of the catalyst.

Click chemistry (CuAAC) and cross-coupling methodologies provide superior regioselectivity, consistently high yields, and reliable performance, making them powerful tools for late-stage functionalization. Their principal limitation lies in the need for prefunctionalized substrates, such as alkynes or halides, which increases synthetic complexity and may limit rapid analogue generation. Fluoroallylation and *gem*-difluorination using silylated fluorinated reagents (*e.g.*, acetyltrimethylsilane, TFMTMS) afford access to structurally distinctive monoterpenoid derivatives while preserving olefinic functionality; however, these multistep strategies are largely confined to acyclic systems and require careful control of reaction conditions to suppress competing side reactions.

Direct fluorination methods highlight the inherent trade-off between reactivity and selectivity. Nucleophilic trifluoromethylation with the Ruppert–Prakash reagent (TMSCF_3_) enables efficient CF_3_ incorporation with tunable diastereoselectivity, yet is limited by steric hindrance, reagent instability, and dependence on fluoride activators. Electrophilic fluorination using Selectfluor—including ketone-directed and metal-catalyzed C–H fluorination—permits late-stage, site-selective modification of rigid monoterpenoid scaffolds without prefunctionalization. Nevertheless, these transformations often suffer from modest yields, restricted positional flexibility, and strong dependence on substrate geometry and C–H bond strength. Although Pd-catalyzed fluorination can deliver enhanced regioselectivity with appropriate ligand design and additives, its broader application is curtailed by catalyst cost, narrow substrate scope, and the need for extensive optimization. Overall, no single fluorination strategy is universally optimal; rather, method selection must balance substrate architecture, the desired mode of fluorine incorporation, and tolerance to reaction conditions.

### 2.2. Fluorinated sesquiterpenoids

Sesquiterpenoids are a class of naturally occurring terpenoid compounds composed of 15 carbon atoms (C_15_), typically assembled from three isoprene units (C_5_H_8_). Unlike simple sesquiterpenes, sesquiterpenoids contain additional functional groups—such as hydroxyls, aldehydes, ketones, or epoxides—that significantly expand their chemical diversity and biological activity. Because of their structural complexity and broad pharmacological potential, incorporating fluorine atoms into sesquiterpenoid frameworks has become an important strategy for modulating their physicochemical properties, metabolic stability, and bioactivity.

#### 2.2.1. Heck cross-couplings

Parthenolide (PTL, 81), isolated from *Tanacetum parthenium* (commonly known as feverfew), is a naturally occurring sesquiterpene lactone widely used in the treatment of fever, migraine, rheumatoid arthritis, and various inflammatory conditions. PTL is a neutral, lipophilic lactone with low polarity, characterized by the presence of an α-methylene-γ-lactone ring and an epoxide moiety—both of which can interact with nucleophilic sites on biological macromolecules.^53^

The conversion of α-methylene-γ-butyrolactones into α-alkylidene-γ-butyrolactones *via* metal-catalyzed processes has gained considerable interest due to its relevance in the efficient synthesis of biologically active natural products that contain these structural motifs.^54,55^

**Scheme 14:**
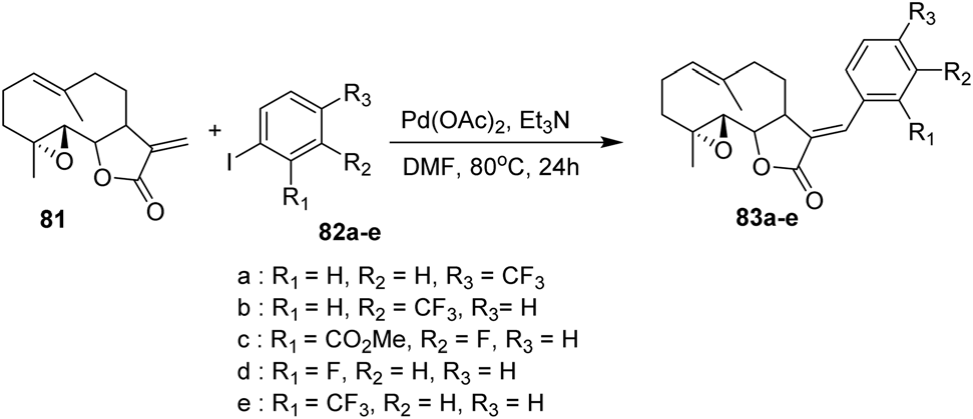
Palladium(ii)-catalyzed Heck-type coupling of parthenolide (81) to afford α-benzylidene-γ-lactones (83a–e).^56^

In 2009, Han *et al.* demonstrated the application of the Heck reaction to convert parthenolide (81), containing the α-methylene-γ-lactone substructure, into a series of α-benzylidene-γ-lactones (83a–e) with excellent selectivity for the E-olefin geometry (Scheme 14).^56^ This strategy provides α-benzylidene-γ-lactone derivatives in good yields and offers a versatile approach for constructing analogues of natural products bearing the α-methylene-γ-lactone motif. In this transformation, only exocyclic olefin products are obtained because the C11–C13 insertion must occur opposite the C7 proton. The required *β*-hydride elimination is not possible on the same face as the C7 protons, making the formation of the exocyclic olefin the exclusive pathway.^56^

#### 2.2.2. Michael addition reactions

Fluorine substitution in PTL-derived compounds can also be accomplished through Michael addition. Sesquiterpene lactones such as parthenolide (PTL) possess an α-methylene-γ-lactone moiety that serves as an excellent Michael acceptor, readily undergoing conjugate addition with nucleophiles.^57^

**Scheme 15:**
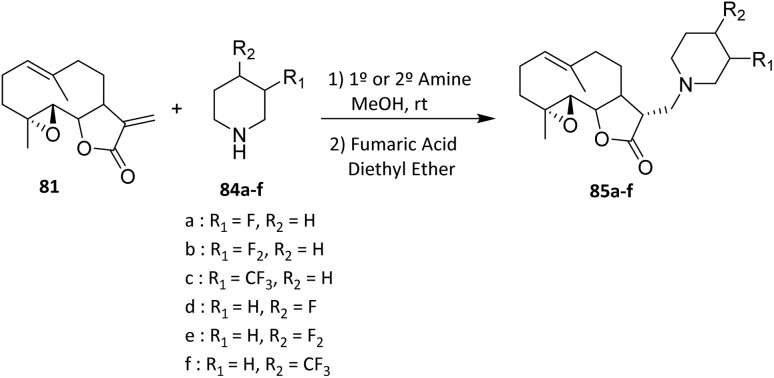
Michael addition (Mannich-type) reaction of parthenolide (PTL) with aliphatic amines to afford amino-parthenolide analogues.^58^

In 2011, Woods *et al.* reported that PTL reacts smoothly with a variety of primary and secondary aliphatic amines to afford the corresponding amino-parthenolide analogues (85a–f) (Scheme 15). The reaction displays broad substrate applicability toward aliphatic amines with varying steric demands, indicating good functional group tolerance under mild conditions. Notably, the electrophilic α,β-unsaturated lactone moiety of PTL undergoes selective Michael addition without competitive epoxide ring opening or scaffold degradation, underscoring the high chemoselectivity of the process. Importantly, the Michael addition was found to be highly stereospecific, delivering exclusively a single stereoisomer bearing the *R*-configuration at C-11. The alternative diastereomer featuring the *S*-configuration at that stereocenter was not detected in the reaction mixture.^58^ This selectivity is governed by the rigid bicyclic framework of PTL, which enforces facial differentiation of the enone and directs nucleophilic attack to the less hindered face. The absence of the *S*-diastereomer suggests a strong conformational and steric control inherent to the natural product scaffold rather than catalyst-induced selectivity.

#### 2.2.3. Deoxofluorination reaction

**Scheme 16:**
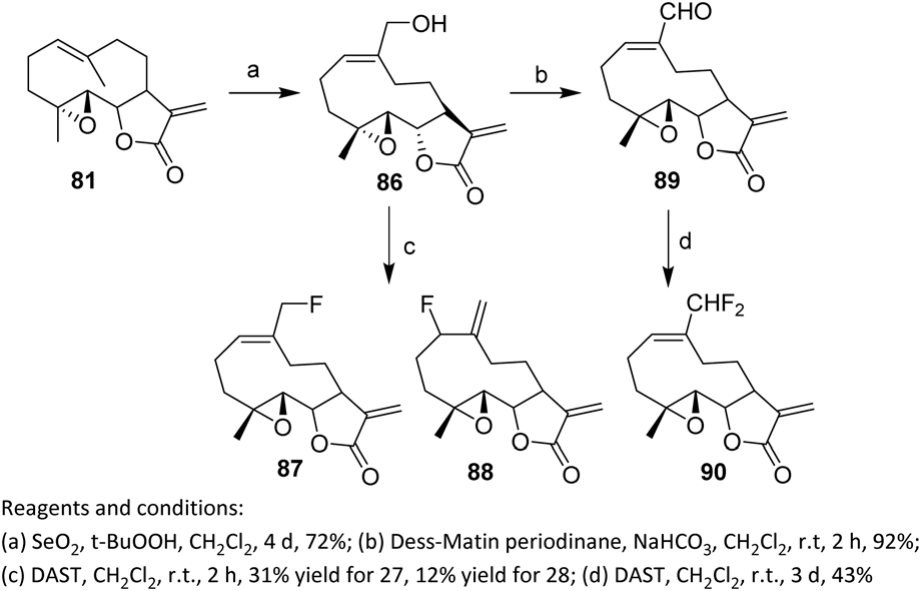
Fluorination with DAST.^60^

The PTL molecule 81 and several of its fluorinated analogs can selectively kill cancer stem cells. Cancer stem cells are defined as cells capable of extensive self-renewal and differentiation into progenitor cells; accumulating evidence indicates that they play key roles in tumor initiation, metastasis, and treatment resistance.^59^ Oxidation of the allylic methyl group of PTL 81 using SeO_2_/t-BuOOH affords alcohol 86 (Scheme 16), demonstrating good chemoselectivity despite the presence of multiple potentially reactive functionalities, including an epoxide and an α,β-unsaturated lactone. This transformation highlights the feasibility of site-selective oxidation on a complex sesquiterpenoid scaffold, thereby providing a versatile handle for subsequent functionalization. Fluorination of 86 with diethylaminosulfur trifluoride (DAST) in CH_2_Cl_2_ produces a 3 : 1 mixture of 87 and 88, which can be separated by recrystallization from diethyl ether to give monofluorinated analogs 87 (31% yield) and 88 (12% yield). The observed 3 : 1 diastereomeric ratio reflects competing reaction pathways governed by local steric and conformational constraints within the rigid PTL framework. Although recrystallization allows separation of the individual isomers, the modest overall yields highlight a key limitation of DAST-mediated fluorination when applied to structurally complex natural products. Furthermore, oxidation of 86 with Dess–Martin periodinane in the presence of NaHCO_3_ furnishes aldehyde 89, which reacts with DAST to give the difluorinated analog 90 in 43% yield.^60^ This result suggests that carbonyl substrates offer a more predictable and efficient route for fluorine incorporation compared to secondary alcohols.

In 2022, Adekenov *et al.* carried out the fluorination of the sesquiterpene grossheimin 91 using DAST. The presence of an easily acylated secondary hydroxyl group drives grossheimin toward fluorination. The reaction of grossheimin 91 with DAST was performed in methylene dichloride and produced a complex mixture, from which two compounds, 92 and 93, were isolated in low yields (3% and 5%, respectively).

**Scheme 17:**
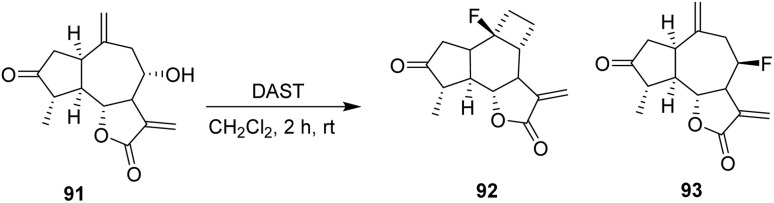
DAST-mediated deoxyfluorination of grossheimin.^61^

The skeletal rearrangement leading to 92 from 91 is triggered by nucleophilic attack of the 8-hydroxyl group of grossheimin on the hypervalent sulfur center of DAST. The activated hydroxyl can then be directly attacked by a fluoride anion to form 93, or alternatively rearrange to a non-classical cyclopropylmethyl cation through nucleophilic attack from the C10(14) exomethylene. Following a formal Demjanov-type ring expansion, the resulting cyclobutyl cation is ultimately trapped by a fluoride anion (Scheme 17).^61^

#### 2.2.4. Click chemistry

Click chemistry is an important approach for the synthesis of drug-like 1,2,3-triazole compounds and has become one of the most rapid and popular strategies for generating compound libraries. Employing click-chemistry methodologies can accelerate the drug-discovery process, since these relatively facile synthetic procedures are typically insensitive to oxygen and water, use readily available reagents, and allow for simple isolation processes.^62^

The synthesis of several MMB–1,2,3-triazole fluorinated derivatives has been reported *via* reaction of a C-14 azido derivative of MMB with a variety of aryl-fluorinated reagents through click-chemistry protocols. In 1982, Feraly reported melampomagnolide B (MMB) 86, an anticancer sesquiterpene lactone isolated from *Magnolia grandiflora*,^63^ a plant that produces several potent sesquiterpene-lactone derivatives active against both hematological and solid tumors.^64^

**Scheme 18:**
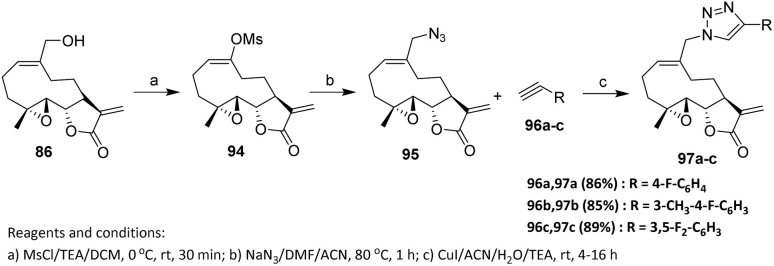
Fluorination *via* click chemistry process.^65^

The MMB mesylate 94 was synthesized by reacting MMB with methanesulfonyl chloride in dichloromethane at 0 °C for 30 min. Compound 94 was then treated with sodium azide in the presence of dimethylformamide and acetonitrile at 80 °C for 1 h to afford the azido synthon 95. Intermediate 95 was subsequently reacted with a series of aryl-fluorine reagents (96a–c) in the presence of CuI/triethylamine/acetonitrile–water (9 : 1) at ambient temperature to give the corresponding MMB-triazole derivatives 97a–c (Scheme 18).^65^ The CuAAC reaction proceeds efficiently under ambient conditions, furnishing triazole-linked products with high regioselectivity, which underscores the inherent robustness of click chemistry. Notably, aryl-fluorine substituents do not interfere with copper catalysis, demonstrating excellent functional group tolerance and enabling late-stage incorporation of fluorinated aromatic motifs.

#### 2.2.5. Electrophilic fluorinating reagents

Decalone 98 is a sesquiterpene with a structure based on the hypothetical drimane skeleton. These compounds play important ecological roles and exhibit various biological activities, including antiviral [influenza A (H1N1)], anti-inflammatory, cytotoxic, and particularly antifeedant properties.^66^

Hydrogen/fluorine interchange at the C-9 position of the decalone system was effected by electrophilic fluorination of the sodium enolate of β-keto ester 98, generated by treatment of 98 with sodium hydride in THF. The efficiency and stereoselectivity of this electrophilic fluorination depend on the nature of the fluorinating agent. The best results were achieved with *N*-fluorobenzenesulfonimide (NFSI), which stereoselectively afforded fluorodecalone 99 in 85% yield, likely due to its moderate electrophilicity and controlled fluorine transfer, which favor attack on the less hindered enolate face. This outcome implies the involvement of a well-organized transition state, dictated by the steric and conformational constraints of the decalone framework.

**Scheme 19:**
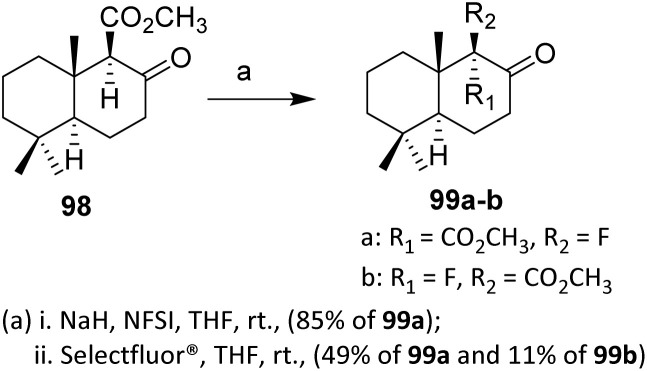
Electrophilic fluorination of decalone.^67,68^

In contrast, fluorination with Selectfluor results in diminished stereocontrol, affording a mixture of the 9-epimeric fluorodecalones 99a and 99b in a combined yield of 65% after chromatographic separation (Scheme 19). The higher electrophilicity and less discriminating fluorine delivery associated with Selectfluor likely favor faster, less ordered reaction pathways, thereby reducing facial selectivity during enolate fluorination. This comparison clearly illustrates that reagent choice plays a decisive role in governing diastereoselectivity in electrophilic fluorination reactions.^67,68^

**Scheme 20:**
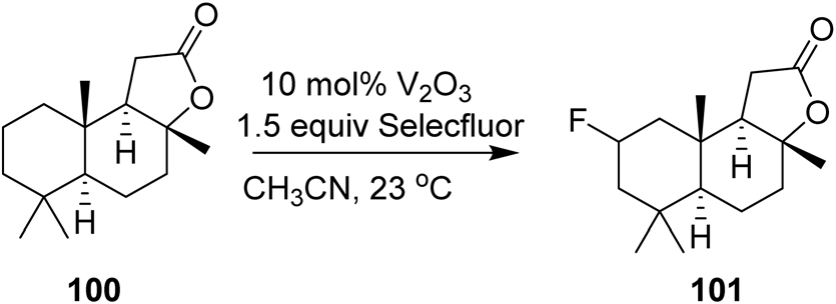
Fluorination of sclareolide with Selectfluor.^50^

Vanadium(iii) oxide catalyzes the direct fluorination of C(sp^3^)–H groups using Selectfluor. This reaction is operationally simple, and both the catalyst and reaction by-products can be easily removed by filtration. In 2014, Xia *et al.* reported that vanadium(iii) oxide catalyzed the fluorination of the sesquiterpenoid sclareolide 100 with improved efficiency and selectivity compared to the manganese–porphyrin catalyst system. The C-2 fluoride 101 (α: *β* = 9 : 1) was obtained in 61% yield, along with the C-3 α-fluoride in 15% yield (C-2: C-3 = 4 : 1), isolated as an inseparable mixture of isomers (Scheme 20).^50^ The concurrent formation of C-3-fluorinated products, albeit in minor amounts, indicates that regioselectivity is not absolute. This observation is consistent with a radical-type mechanism, in which hydrogen abstraction preferentially occurs at weaker or more accessible C–H bonds while still permitting competitive fluorination at adjacent positions. The isolation of the products as an inseparable mixture further underscores the inherent limitations in achieving precise site- and stereoselective control in direct C–H fluorination reactions.

The bicyclic sesquiterpene (+)-valencene 102 is a constituent of the essential oils of various *Citrus* species, including sweet orange (*Citrus sinensis*).^69^ The bicyclic sesquiterpenoid (+)-valent 95 contains a ketone functional group in its structure, which can be used to direct aliphatic fluorination using Selectfluor, catalytic benzil, and visible light. Bume *et al.* reported a visible light-sensitized ketone-directed C–H fluorination method employing catalytic benzil (10 mol%), Selectfluor (as a putative atomic fluorine source),^47^ and cool white LEDs.^48^ Under these mild conditions, predictable *β*- or *γ*-fluorination can be achieved depending on the proximity of the C–H bond to the ketone.

**Scheme 21:**
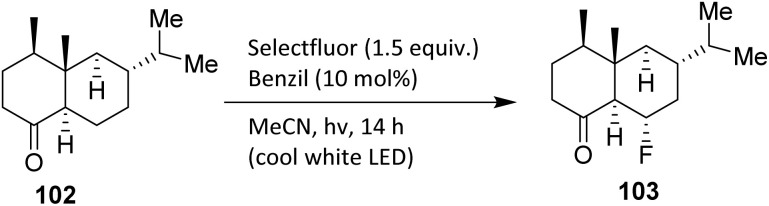
C–H fluorination of valencene using a benzil photocatalyst.^49^

It is noteworthy that other N–F reagents, such as NFSI and *N*-fluoropyridinium tetrafluoroborate, failed to generate the desired fluorinated product 103 (Scheme 21). Although NFSI can react with alkyl radicals, Selectfluor reacts at a significantly faster rate and is more likely to participate in electron-transfer processes. For example, compound 103 (fluorinated from the sesquiterpenoid valencene) was formed selectively, despite the presence of other tertiary carbon sites more distant from the ketone.^49^

Fluorination of sesquiterpenoid derivatives 104 can also be achieved using a palladium catalyst. As with other palladium-catalyzed fluorination reactions employing fluoride salts, control of regioselectivity remains a key challenge in developing a practical synthetic method.^51^ The addition of triethyl(trifluoromethyl)silane (TESCF_3_) was found to effectively overcome this issue, leading to dramatically improved regioselectivity in the palladium-catalyzed fluorination process.

**Scheme 22:**
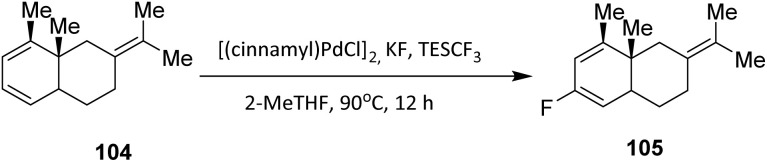
Fluorination of sesquiterpenoids derivatives 104*via* a palladium-catalyzed reaction.^52^

This finding, together with the use of a newly developed biarylphosphine ligand, enabled the establishment of an efficient and highly regioselective protocol for the fluorination of monoterpenoid derivatives. Under these optimized conditions, the desired fluorinated product 105 was obtained in 41% yield (Scheme 22).^52^

#### 2.2.6. Comparative summary of fluorination strategies for sesquiterpenoids

Fluorination strategies applied to sesquiterpenoids encompass a broad spectrum of reactivity, ranging from scaffold modification to direct C–F bond formation, each presenting distinct advantages and limitations. Transformations such as Heck cross-coupling and Michael addition are particularly effective for the functionalization of α-methylene-γ-lactone–containing sesquiterpenoids, exemplified by parthenolide. These reactions proceed under relatively mild conditions and offer high chemo- and stereoselectivity while preserving the integrity of the complex sesquiterpenoid framework. However, fluorine incorporation in these approaches is typically indirect, relying on fluorinated coupling partners or nucleophiles, which limits their ability to modulate the intrinsic electronic properties of the sesquiterpenoid core.

Deoxofluorination and click chemistry provide complementary avenues for introducing fluorine into sesquiterpenoid derivatives. DAST-mediated deoxofluorination enables the direct replacement of hydroxyl or carbonyl functionalities with fluorine, granting access to mono- and difluorinated analogues. While carbonyl-containing substrates generally afford higher efficiency, reactions involving secondary alcohols often deliver only moderate yields, suffer from competing rearrangements, and display limited stereochemical control. In contrast, CuAAC click chemistry allows rapid and regioselective attachment of fluorinated aromatic moieties *via* triazole linkers, exhibiting excellent functional group tolerance and suitability for late-stage diversification. Nevertheless, this strategy appends fluorinated units externally rather than modifying the carbon framework of the sesquiterpenoid itself.

Direct electrophilic and catalytic fluorination methods further illustrate the delicate balance between reactivity and selectivity in structurally complex sesquiterpenoid systems. Electrophilic fluorination using reagents such as NFSI or Selectfluor enables direct C–F bond formation at enolate or activated C–H positions, with NFSI generally providing superior stereocontrol, whereas Selectfluor offers higher reactivity at the expense of selectivity. Metal- and photocatalyzed C–H fluorination strategies permit late-stage, site-selective modification without prior functionalization; however, they often generate regioisomeric mixtures and afford only modest yields. Palladium-catalyzed fluorination can improve regioselectivity through appropriate ligand and additive selection, yet its broader application is limited by catalyst cost, narrow substrate scope, and the need for extensive optimization. Collectively, these observations highlight that no single fluorination strategy is universally optimal; instead, method selection must be guided by the specific sesquiterpenoid scaffold, the desired mode of fluorine incorporation, and the tolerance of the system to the required reaction conditions.

### 2.3. Fluorinated diterpenoids

Nature produces an impressive assortment of more than 3300 known diterpenoids, many of which are specialized (secondary) metabolites of plant origin. Diterpenoids are widely distributed throughout the plant kingdom and have long been recognized for their broad spectrum of biological activities. For example, isopimaric acid 102 is a readily available and versatile tricyclic diterpenoid, abundantly found in the resin of conifer genera such as *Pinus*, *Larix*, and *Picea*.^70^

#### 2.3.1. The cross-coupling–cyclization reaction (Pd catalsyt)

The design and synthesis of novel fluorine-substituted isopimaric acid derivatives provide a promising approach to access new molecules with enhanced anticancer activity. Gromova *et al.* employed readily available derivatives, namely *N*-(2,3-butadienyl)carboxamide of isopimaric acid 106 and fluorinated 2-iodophenols 107, whose syntheses have been reported previously.^71^

The main synthetic interest in allenes lies in the formation of carbo- and heterocycles. Pd-catalyzed cross-coupling of allenes with aromatic iodides bearing a nucleophilic substituent at the *ortho* position (*e.g.*, 2-iodoaniline and 2-iodophenol) allows cyclization *via* [*α*,*β*], [*β*,*α*], [*β*,*γ*], or [*γ*,*β*] attack on the allene, resulting in the formation of indole or benzofuran frameworks.

**Scheme 23:**
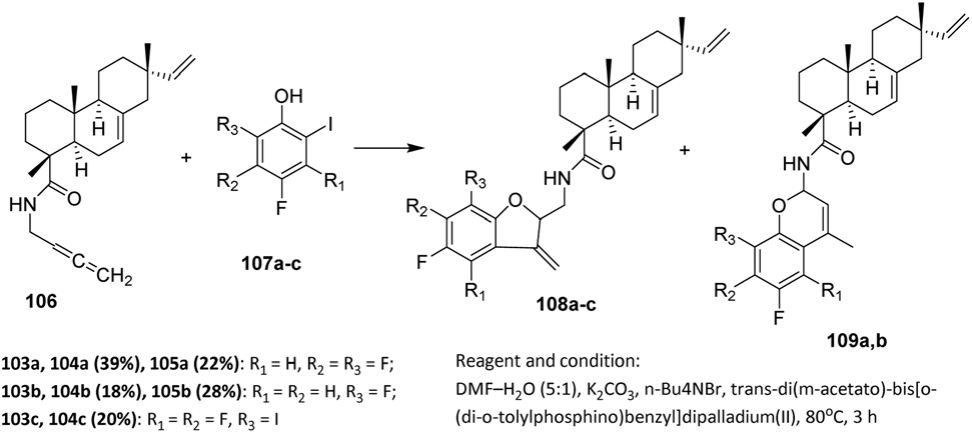
Synthesis of furo[3,2-*c*]chromen-4-one derivatives *via* Pd-catalyzed coupling-cyclization.^72^

Experimental results by Gromova *et al.* demonstrated that the nature of the substituent in the fluorinated phenols significantly influences the outcome of the cross-coupling–cyclization reaction. Mechanistically, and according to the principles of palladium catalysis, the formation of compounds 108 and 109 can be described as follows (Scheme 23).

The pallacycle catalyst is reduced to a Pd(0) species under the reaction conditions (DMF–H_2_O, K_2_CO_3_, Bu_4_NBr, fluorine-substituted 2-iodophenol, allene). The Pd(0) species is generated *via* nucleophilic attack of the fluorine-substituted 2-iodophenol on the palladacycle, forming an arylated palladacycle that undergoes reductive elimination to yield the active Pd(0) catalyst.

Subsequently, a nucleophilic attack by the substituent at the α-position leads to the expected products 108. The formation of isomeric compounds 109 involves generation of an *η*^3^-allylpalladium intermediate, in equilibrium with the less stable σ-vinylpalladium species, followed by *η*^1^-rearrangement, *β*-elimination, and attack on the activated double bond. The substituent on 2-iodophenols 107a–c affects the stabilization of reaction intermediates, thereby influencing the selective formation of 108 or 109.^72^

#### 2.3.2. Acylation of the amino group

Extensive studies on the structure–activity relationship of docetaxel have mainly focused on modifications of the C-13 side chain and the B and C rings. The importance of the C-13 substituted phenylisoserine side chain for the bioactivity of docetaxel has long been recognized. Fluorination of docetaxel is expected to impact its metabolic stability and pharmacokinetic profile.^73^

Lu *et al.* reported the synthesis, cytotoxic activity, metabolic stability, and pharmacokinetics of a series of fluorinated docetaxel analogs. First, the hydroxyl groups at C-7 and C-10 of 10-deacetylbaccatin III (10-DAB) 109 were protected with 2,2,2-trichloroethyl chloroformate (TrocCl) using pyridine as the base, affording 7,10-diTroc-10-deacetylbaccatin 110. Compound 110 was then coupled with 111 in the presence of dicyclohexylcarbodiimide (DCC) and 4-dimethylaminopyridine (DMAP) to give intermediate 112 in 86% yield. The high yield obtained for intermediate 112 indicates that carbodiimide activation remains effective even in complex diterpenoid systems, provided that reactive hydroxyl groups are appropriately masked.

**Scheme 24:**
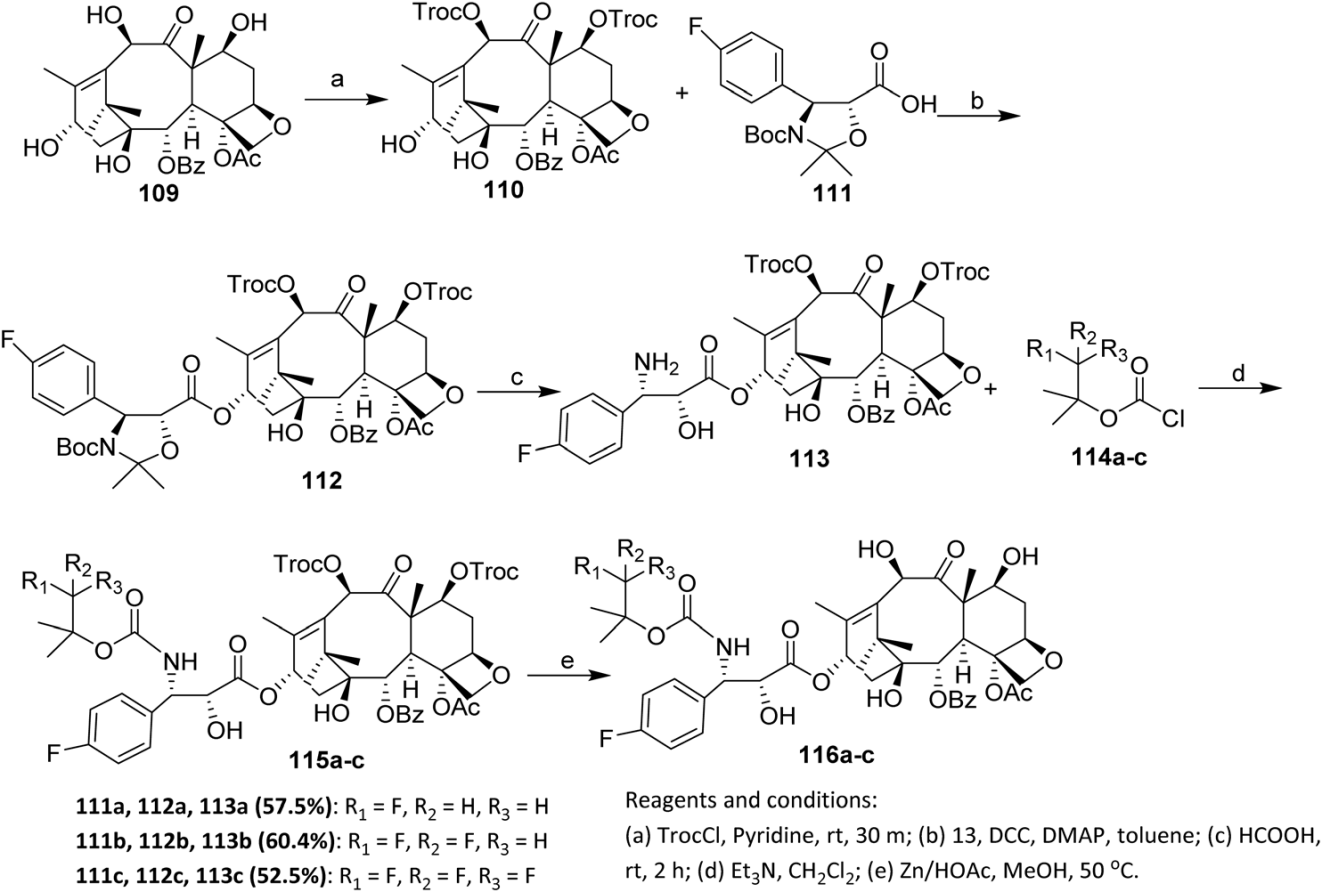
Fluorination *via* acylation of the amino group.^25^

After removal of the 30-*N*-boc and acetonide protecting groups from 112 using 98% formic acid at room temperature, the amino alcohol 113 was obtained in 55% yield. Subsequent acylation of the amino group of 113 with freshly prepared fluorine-containing acyl chlorides 114a–c, followed by removal of the 7,10-troc protecting groups with zinc in acetic acid, furnished the corresponding fluorinated docetaxel derivatives 116a–c in desirable yields (Scheme 24).^25^ Despite its effectiveness, this approach is inherently multistep and relies heavily on protecting-group manipulations, which may limit scalability and overall synthetic efficiency. Moreover, fluorine is introduced indirectly through side-chain modification rather than by direct fluorination of the taxane core, thereby restricting structural diversification of the diterpenoid backbone.

#### 2.3.3. Copper(0)-catalysis

Abietanes are a family of naturally occurring tricyclic diterpenoids that have been isolated from various terrestrial plants. These compounds have attracted significant synthetic interest due to their wide range of biological activities, including antimicrobial, antifungal, antiprotozoal, and anticancer properties.^74^

**Scheme 25:**
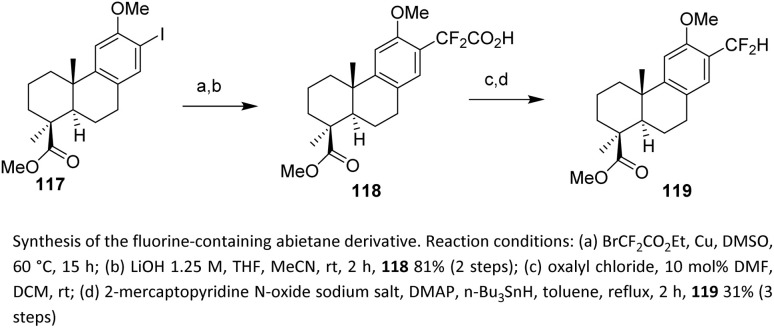
Synthesis a fluorine-containing abietane derivative *via* copper(0) catalysis.^75^

In 2019, Lapuh *et al.* reported the synthesis of a fluorine-containing abietane derivative *via* a copper(0)-catalyzed process. The use of a Cu(0) catalyst enables efficient installation of a difluoroacetate fragment onto an iodoarene precursor, providing a practical entry point for the incorporation of fluorinated carbon units into aromatic terpene-derived systems. Starting from iodoarene 117, the ethyl difluoroacetate moiety was efficiently introduced through a copper(0)-mediated coupling in DMSO. The choice of DMSO as coordinating polar aprotic solvent likely facilitates copper-mediated oxidative addition and stabilizes reactive intermediates, contributing to the efficiency of the C–C bond-forming step. Subsequent selective saponification of the ethyl ester afforded carboxylic acid 118 in 81% yield. This step underscores the stability of the CF_2_ group under basic hydrolytic conditions and enables further functional manipulation. A three-step sequence including a Barton decarboxylation then produced the difluoromethyl derivative 119 (Scheme 25).^75^

#### 2.3.4. Electrophilic fluorination

Interest in kaurane diterpenoids has recently increased, as some of them exhibit diverse biological activities, including antimicrobial, anti-inflammatory, anti-HIV, cytotoxic, antifertility, and insect antifeedant effects. Kauranoids are also known as biological precursors of gibberellins, plant growth-regulating hormones.

**Scheme 26:**
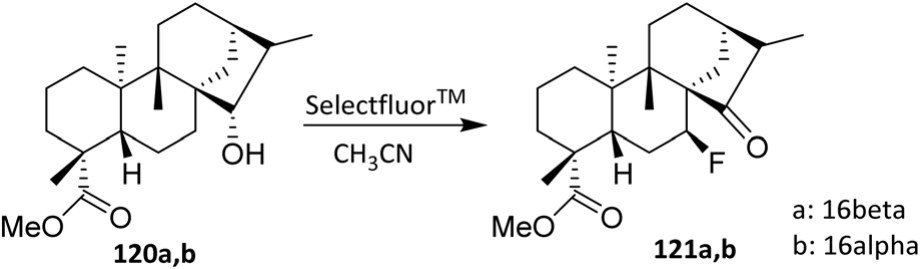
Fluorination of hydroxykauranes using Selectfluor.^76^

The fluorination of hydroxykauranes 120 likely begins with the formation of an intermediate hypofluorite, which acts as an electrophilic fluorinating reagent. In the case of the 15*α*-epimers 120a and 120b, the fluorine atom of the hypofluorite can access the equatorial H-7*α*, abstracting a hydrogen atom to form HF (captured by an amine) and oxidizing C-15 to generate an intermediate ketone in an enthalpy-favored process. This intermediate then reacts with a complex amine·HF (similar to commercial TREAT HF) from the less hindered side (opposite H-7*α*) to afford the fluoroderivatives 121a (10%) and 121b (12%) (Scheme 26).^76^ Nucleophilic attack occurs preferentially from the less hindered face of the molecule, opposite to H-7*α*, leading to the observed fluoroderivatives. This sequence illustrates that stereochemical outcome is dictated primarily by substrate topology rather than by reagent control.

#### 2.3.5. Acylation of the hydroxyl group

Oridonin 122 is a widely distributed *ent*-kaurene found in *Rabdosia* plants and has recently attracted significant attention due to its anti-tumor activity, particularly its novel mechanism of inhibiting NF-κB activation.^77^ In 2016, Li *et al.* reported the fluorination of oridonin *via* an acylation approach. Compound 124 was synthesized from 122 by selective oxidation using Jones reagent, with no further purification required for the subsequent step.

**Scheme 27:**
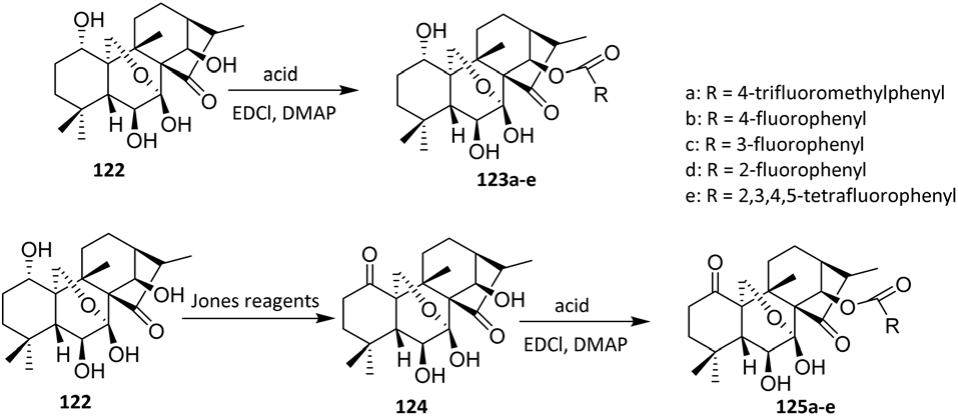
Fluorination of oridonin *via* an acylation method.^78^

This step demonstrates that the diterpenoid framework tolerates strong oxidative conditions without degradation, thereby enabling a streamlined synthesis without intermediate purification. The target compounds 123a–e and 125a–e were obtained by treating 122 or 124 with the corresponding acid in the presence of 1-ethyl-3-(3-dimethylaminopropyl)carbodiimide (EDCI) and 4-dimethylaminopyridine (DMAP) in dichloromethane (DCM) at room temperature for 8–12 hours. Under these conditions, the 14-OH group was regioselectively esterified, reflecting its higher nucleophilicity and steric accessibility relative to other potential reactive sites on the oridonin skeleton. This selectivity enables predictable late-stage functionalization of a complex natural product (Scheme 27).^78^

In another study, Bessonova *et al.* (2000) reported the fluorination of Kobusine *via* the acylation method. Kobusine 126 is a hetisine-type C20-diterpenoid alkaloid and a major constituent of *Aconitum yesoense* var. *Macroyesoense*.^79^

**Scheme 28:**
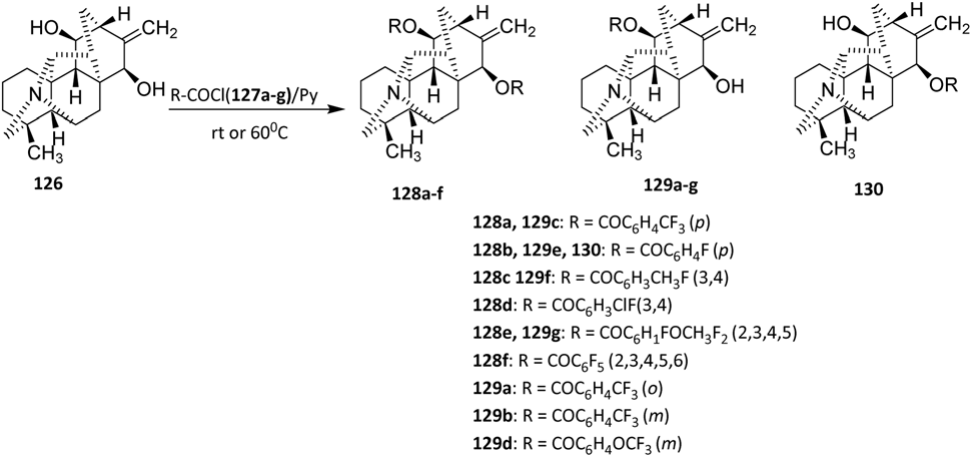
Fluorination of Kobusine *via* acylation method.^80,81^

Kobusine 126 was reacted with various acyl chlorides (127a–g) in pyridine to afford C-11, C-15, or C-11,15-substituted acyl derivatives (128a–f, 129a–g, and 130) (Scheme 28).^80,81^ The formation of C-11, C-15, or C-11,15-disubstituted products reflects differences in the relative nucleophilicity and steric accessibility of the hydroxyl groups present in kobusine. Competition between these sites results in product distributions that are predominantly substrate-controlled rather than catalyst-controlled. While the ability to access multiple substitution patterns indicates a degree of substrate flexibility, it also underscores the limited inherent regioselectivity of the transformation under the selected conditions. This transformation proceeds *via* nucleophilic attack of the hydroxyl group on the carbonyl carbon of the acid chloride. Pyridine acts both as a base to neutralize the released HCl and as a catalyst to facilitate ester bond formation.

**Scheme 29:**
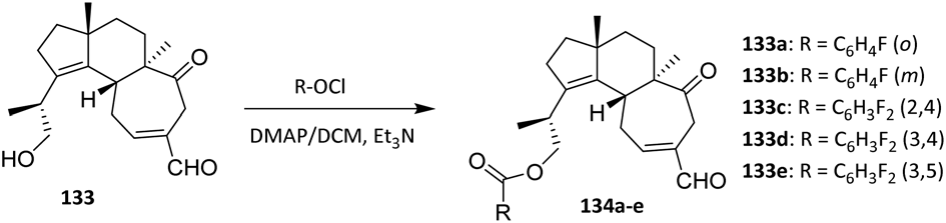
Fluorination of sarcodonin G 133.^82^

Cao *et al.* also performed fluorination of the diterpenoid sarcodonin G 133 using the acylation method. Sarcodonin G 133 was obtained quantitatively from the fruit bodies of *S. scabrosus*. A series of 19-*O*-benzoyl derivatives was prepared as shown in Scheme 29. Briefly, 133 was treated with dry Ac_2_O or the respective benzoyl chloride derivatives in the presence of 4-dimethylaminopyridine (DMAP) at room temperature. Use of DMAP as a nucleophilic acyl transfer catalyst facilitates efficient ester bond formation under mild conditions, minimizing side reactions and preserving the integrity of the polycyclic framework. Workup of the resulting residue provided new compounds (134a–e) as yellowish oils with purity >99.0% by HPLC and in good to excellent yields (Scheme 29).^82^ The acylation reaction proceeds with high regioselectivity at the C-19 hydroxyl group, reflecting its enhanced nucleophilicity and steric accessibility relative to other potential reactive sites on the diterpenoid scaffold.

#### 2.3.6. Deoxofluorination reaction

**Scheme 30:**
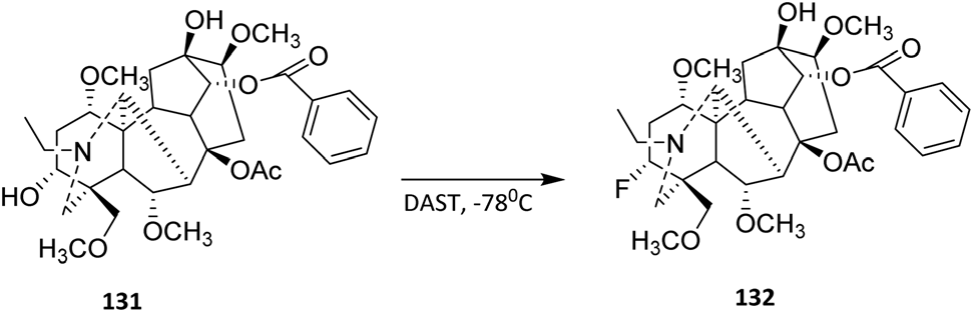
Deoxofluorination of indaconitine 131.^84^

Diethylaminosulfur trifluoride (DAST) is a versatile reagent widely used for the deoxofluorination of alcohols and carbonyl compounds. In 2023, Zhang *et al.* conducted the deoxofluorination of indaconitine 131. Aconitine-type diterpenoid alkaloids have received widespread attention due to their potent anti-tumor activities.^83^ The hydroxyl groups in 131 were selectively replaced by fluorine atoms. This transformation is valuable in medicinal and materials chemistry, as fluorinated molecules often exhibit enhanced stability and bioactivity. The substrate scope illustrated by this transformation underscores the applicability of DAST to complex, polyoxygenated natural products. Despite the presence of several hydroxyl functionalities in indaconitine, selective activation of a single secondary alcohol was achieved, affording fluorinated derivative 132 in 58% yield (Scheme 30). This result suggests that DAST is compatible with rigid polycyclic systems and can be employed for late-stage fluorination, a desirable feature in medicinal chemistry for fine-tuning biological and physicochemical properties.^84^

Reactivity and selectivity in DAST-mediated deoxofluorination are governed by a combination of steric and electronic factors. More accessible hydroxyl groups and those capable of forming stable alkoxysulfur intermediates are preferentially fluorinated, while sterically hindered or intramolecularly hydrogen-bonded OH groups are less reactive. In indaconitine, the conformational exposure of the 3-OH in the A-ring likely facilitates its activation by DAST, whereas other hydroxyl groups remain unreacted under the same conditions.

#### 2.3.7. Amide reaction

**Scheme 31:**
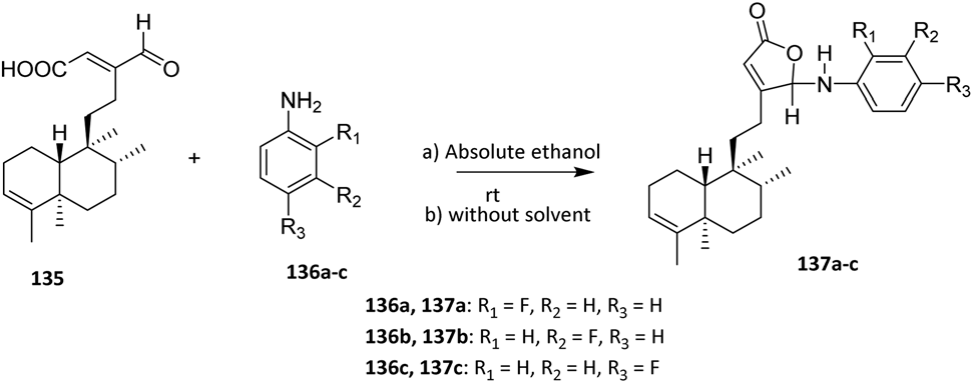
Fluorination of diterpenoid (135) *via* amide reaction.^85^

An amide reaction is a condensation process in which a carboxylic acid (or its derivative) reacts with an amine to form an amide bond, often accompanied by the elimination of water or another small molecule. Scheme 31 represents an amide bond formation between the carboxylic acid group of compound 136 and the aromatic amine group of compounds 137a–c. The condensation proceeds either in absolute ethanol or under solvent-free conditions, giving the amide derivatives 137a–c. This transformation introduces an anilide moiety into the steroid framework, enhancing structural diversity and potential bioactivity.

Diterpenoid 135 was reacted in one-pot with 2-fluoroaniline 136a, 3-fluoroaniline 136b, and 4-fluoroaniline 136c in absolute ethanol under catalyst-free conditions at room temperature to yield three new fluorinated diterpenoids 137a–c (Scheme 31). The reaction proceeds without external activation, underscoring the intrinsic reactivity of the diterpenoid scaffold and its suitability for mild, late-stage functionalization.

From a selectivity and substrate-scope perspective, the method tolerates different positional isomers of fluoroaniline, indicating that the electronic effects of the fluorine substituent exert only a limited influence on the overall reaction outcome. However, the longer reaction times observed with fluoroanilines compared to non-fluorinated anilines suggest that the electron-withdrawing fluorine atom reduces aniline nucleophilicity, thereby slowing the transformation.

Reactivity is governed primarily by intramolecular cyclization: *in situ* generation of a reactive intermediate enables nucleophilic attack by the hydroxyl group of the acid moiety, leading to γ-lactone formation. This intramolecular step confers high regioselectivity, as lactonization is dictated by spatial proximity and conformational rigidity of the diterpenoid framework rather than by external catalysts or additives.^85^

**Scheme 32:**
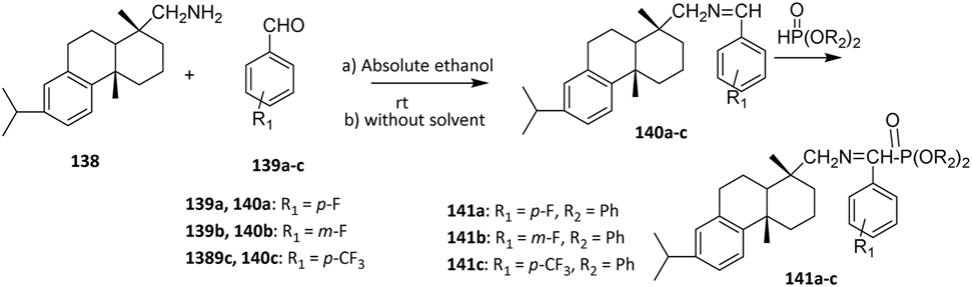
Fluorination of diterpenoid dehydroabietylamine 138.^86^

In another study, Rao *et al.* (2008) reported the fluorination of diterpenoid dehydroabietylamine 138*via* two-step reactions through imine intermediates 140a–c to yield novel α-aminophosphonates 141a–c. Despite the high steric hindrance of the tricyclic structure, dehydroabietylamine 138 and substituted benzaldehydes 139a–c afforded imines in good yields without catalysts. The subsequent addition of phosphites to the imines was challenging due to steric hindrance; benzyl phosphonates gave relatively low yields of 20–30%, whereas ethyl phosphonates provided higher yields owing to less steric congestion (Scheme 32).^86^

#### 2.3.8. Comparative summary of fluorination strategies for diterpenoids

Fluorination and fluorine-based functionalization strategies applied to diterpenoids reflect the pronounced structural complexity and functional diversity inherent to this class of natural products. Cross-coupling–cyclization reactions and metal-mediated C–C bond-forming processes, including Pd-catalyzed allene coupling and Cu(0)-catalyzed difluoroacetate installation, have proven particularly effective for constructing fluorinated polycyclic frameworks. These approaches enable access to novel heterocyclic or aryl-linked motifs with excellent chemo- and regioselectivity while preserving the rigid diterpenoid backbone. Their principal advantages lie in the generation of structural diversity and the availability of innovative scaffolds with potential anticancer activity. However, these methods often require prefunctionalized substrates (*e.g.*, allenes or aryl iodides) and finely tuned catalytic systems, thereby increasing synthetic complexity and potentially limiting scalability.

Acylation strategies, including the acylation of amino or hydroxyl functionalities, represent some of the most reliable and widely applicable methods for introducing fluorinated moieties into diterpenoids such as docetaxel, oridonin, kobusine, and sarcodonin G. These transformations generally proceed under mild conditions with high regioselectivity dictated by the intrinsic nucleophilicity and steric accessibility of specific functional groups. Their advantages include broad functional group tolerance, reproducibility, and suitability for late-stage modification of bioactive compounds. In these cases, however, fluorine is incorporated indirectly *via* fluorinated acyl substituents rather than through direct C–F bond formation on the diterpenoid core. In multistep systems such as taxanes, extensive protecting-group manipulations further reduce synthetic efficiency and constrain the rapid generation of analogues.

Direct fluorination approaches, including electrophilic fluorination and DAST-mediated deoxofluorination, enable the direct installation of C–F bonds, thereby exerting a more pronounced influence on electronic properties, metabolic stability, and biological activity. Electrophilic fluorination of hydroxykauranes illustrates that stereochemical outcomes are largely governed by substrate control, dictated by molecular topology rather than reagent identity, although yields are often modest. DAST-mediated deoxofluorination constitutes a valuable late-stage strategy for highly functionalized diterpenoids, allowing selective fluorination of accessible hydroxyl groups. Nevertheless, such reactions may suffer from limited regioselectivity, competing side reactions, and sensitivity to steric and conformational constraints. Complementary amide-based transformations further expand structural diversity but are often limited by the reduced nucleophilicity of fluoroanilines. Overall, no single fluorination strategy is universally optimal; instead, the choice of method must balance structural complexity, desired fluorine placement, reaction efficiency, and compatibility with the diterpenoid framework.

### 2.4. Fluorinated triterpenoids

Triterpenoids are one of the largest classes of natural products, with over 20 000 known members. They are biosynthesized in plants *via* cyclization of squalene. Over the last decades, pentacyclic triterpenoids have shown a unique range of pharmacological activities, including chemopreventive and antineoplastic potential.^87–91^

#### 2.4.1. Electrophilic fluorination

Selectfluor is a crystalline white solid that is thermally stable and easy to handle because its by-products are usually removed readily with aqueous workup. The reaction of alkenes with Selectfluor typically requires a nucleophilic donor, and the choice of solvent, temperature, and nucleophile can influence the efficiency, regioselectivity, and number of byproducts.^92^

**Scheme 33:**
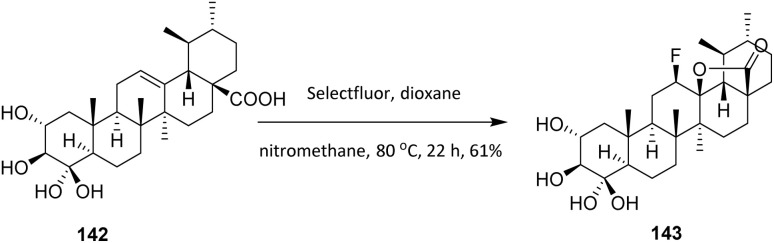
Fluorination of asiatic acid (142) using Selectfluor.^92^

Asiatic acid (AA, 142), a member of the ursane family extracted mainly from *Centella asiatica*, exhibits hepatoprotective, neuroprotective, antidiabetic, antihyperlipidemic, anti-inflammatory, antioxidant, and anti-Alzheimer activities. Goncalves *et al.* (2016) used Selectfluor to introduce a fluorine atom into AA to increase its anticancer activity. Treatment of 142 with Selectfluor in a mixture of nitromethane and dioxane at 80 °C afforded the 12*β*-fluorolactone derivative 143 in 61% yield (Scheme 33). This transformation highlights the high regio- and stereoselectivity of Selectfluor, as fluorination occurs exclusively at the activated C-12 position to give the 12*β*-fluorolactone, reflecting strong substrate control imposed by the lactone framework and neighboring functional groups. While the method exhibits good efficiency for this specific diterpenoid scaffold, its substrate scope appears limited, as elevated temperatures (80 °C) are required and overoxidation or competing electrophilic reactions may occur in more densely functionalized or sensitive analogues.

**Scheme 34:**
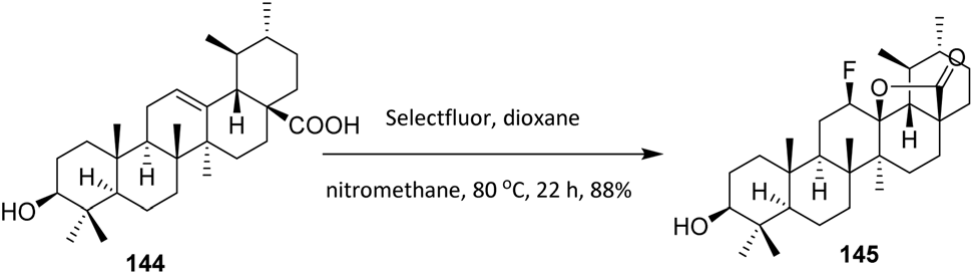
Fluorination of ursolic acid (144) using Selectfluor.^94^

In a different study, Leal *et al.* (2018) developed fluorinated ursolic acid 144 derivatives in an effort to further improve their antitumor properties activity. Fluorine was introduced into the C ring of ursolic acid 145. Ursolic acid 144 is a pentacyclic triterpenoid present in plants, vegetables and fruits, which has been found to have many biological properties, such as anti-inflammatory, antidiabetic, cardioprotective, hepactoprotective, antitumoral and chemopreventive activities.^93^ The fluorination of ursane-type triterpenoids, when performed in a reaction solution without a nucleophilic donor, allows the synthesis of a fluorolactone. The free acid behaves like the nucleophilic donor, losing the proton and promoting cyclization to the carbon C13 in the ursane backbone. This reaction is not possible in ursane-type compounds where the acid is protected, however, as the free acid alone does not react with Selectfluor. Compound 144 was reacted with Selectfluor at 80 °C in a mixture nitromethane and dioxane, produced 145 with the insertion of *β*-fluorine at C12 with a yield above 88% (Scheme 34).^94^

**Scheme 35:**
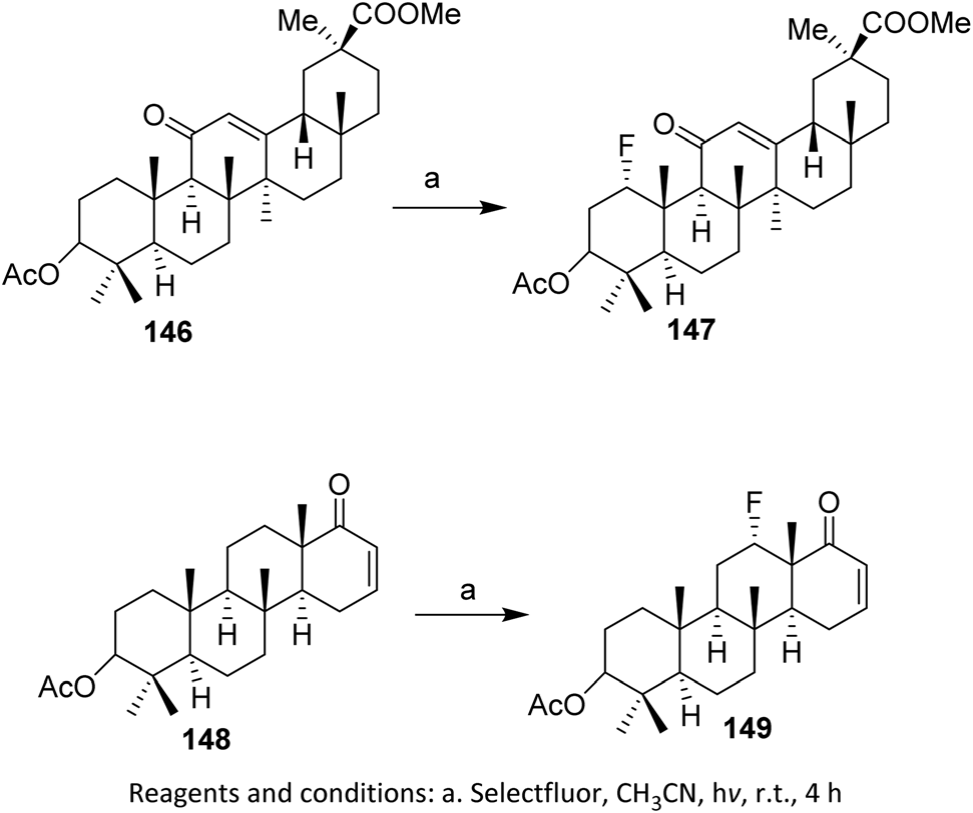
Photoinduced C–H fluorination with Selectfluor.^97,98^

In a separate study, compound 142, a pentacyclic triterpenoid 18-*β*-glycyrrhizinate with an enone on the C-ring, was fluorinated by Pitts *et al.* (2017). Fluorination at the C1 position afforded the product 143 in good yield (up to 72%).^95,96^ This selectivity is governed by the intrinsic polarization of the α,β-unsaturated carbonyl system, which directs electrophilic fluorine toward the more electron-rich and sterically accessible site. In addition to enones targeting the *γ* position, Bume *et al.* (2018) performed enone fluorination at the *β* position. Compound 144, containing an enone in ring D, was fluorinated to give 145 in 70% yield (Scheme 35), highlighting that the reaction outcome is sensitive to both the enone position and the ring topology within the pentacyclic scaffold.^97,98^ The reactivity and selectivity of these transformations are governed by conjugation effects within the enone system, steric shielding imposed by the rigid triterpenoid framework, and the strong electrophilicity of Selectfluor. Collectively, these factors favor controlled monofluorination while suppressing over-fluorination and competing side reactions, even in highly functionalized substrates.

**Scheme 36:**
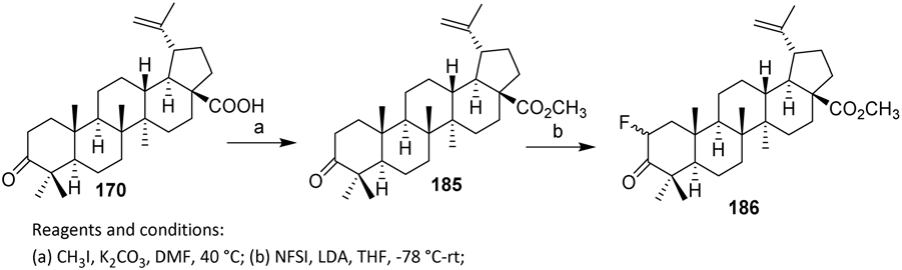
Electrophilic fluorination of betulinic acid derivatives.^99,100^

Li *et al.* introduced fluorine atoms directly at the C-2 side chain of 185 as shown in Scheme 36. Methyl ester 185 was prepared from betulonic acid 170 following reported methods. Fluorination was achieved by treating 185 with lithium diisopropylamide (LDA) and *N*-fluorobenzenesulfonimide (NFSI) at −78 °C, resulting in the addition of one fluorine atom at C-2 to afford 186 in 43% yield *via* an electrophilic substitution reaction.^99,100^ The selection of NFSI as a mild and selective electrophilic fluorine source is critical, as it promotes single fluorine transfer while suppressing over-fluorination and oxidative side reactions. The use of low temperature further stabilizes the enolate and minimizes enolate equilibration, thereby contributing to the observed site selectivity. With respect to substrate scope, this strategy is best suited to triterpenoids bearing acidic α-protons adjacent to carbonyl functionalities, such as esters or ketones, and thus suggests potential applicability to other betulonic acid derivatives. However, substrates lacking enolizable positions or containing base-sensitive functional groups may be incompatible.

**Scheme 37:**
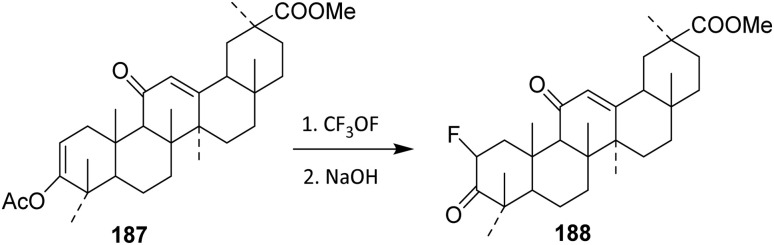
Fluorination of steroidal compound using CF_3_OF reagent.^101^

Trifluoromethyl hypofluorite (CF_3_OF) was applied to methyl olean-2,12(13)-dien-ll-on-30-oat-3β-yl acetate 187. Under sufficiently mild conditions, only the enol acetate system reacts, while the double bond at the 12(13) position remains unaffected. This selectivity reflects the strong peference of CF_3_OF for activated enol systems adjacent to carbonyl groups, where electrophilic fluorine transfer is kinetically favored. Scheme 37 shown an electrophilic fluorination using trifluoromethyl hypofluorite (CF_3_OF) as the fluorinating agent. In this process, CF_3_OF functions as a highly potent electrophilic fluorinating agent, delivering fluorine to the α-position of the carbonyl *via* an enol or enol acetate intermediate. Subsequent base treatment is essential, as it promotes elimination and rearrangement to afford the thermodynamically more stable methyl 2α-fluoroolean-12-ene-3,11-dion-30-oate 188, thereby completing the functional group interconversion in a controlled manner.^101^ The limitations of this methodology include the reliance on CF_3_OF, a highly reactive and potentially hazardous reagent that necessitates careful handling and strictly controlled reaction conditions.

**Scheme 38:**
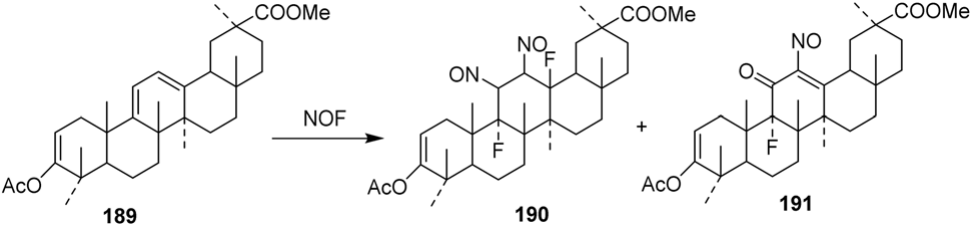
Simultaneous nitration–fluorination of a steroidal derivative.^103^

Nitrosyl fluoride (NOF) generally adds to olefinic bonds to give fluoro-nitrimino compounds, which decompose on alumina to fluoroketones.^102^ However, the reaction of NOF with dienes has not been widely reported. An excess of NOF with methyl 3-*O*-acetyl-9,11-dehydro-11-deoxo-glycyrrhetate 189 in methylene chloride (10 days at 5 °C) gave a mixture, which upon separation by thin-layer chromatography (TLC) afforded 190 (10%) and 191 (25%) (Scheme 38).^103^ Unlike its more common reactions with isolated olefins, NOF engages the diene only under prolonged, low-temperature conditions, indicating low intrinsic reactivity that necessitates the use of excess reagent and extended reaction times. From a selectivity and mechanistic perspective, the formation of two separable products (190 and 191) points to competing regio- and/or stereochemical pathways during electrophilic addition across the conjugated system. The modest yields further suggest that multiple addition modes or parallel decomposition pathways operate concurrently, thereby diminishing overall efficiency and reaction control.

#### 2.4.2. Deoxofluorination reaction

**Scheme 39:**
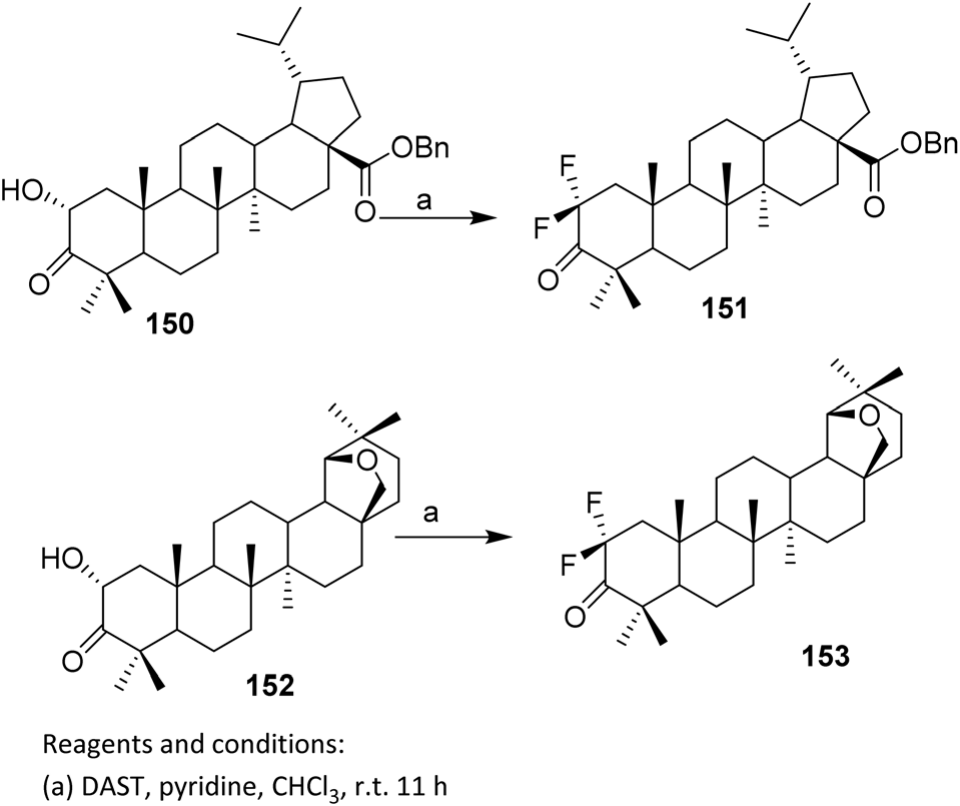
Fluorinateion of triterpenoid *via* deoxofluorination reaction.^24,104^

Benzyl-2α-hydroxydihydrotruelonate 150 was treated with DAST in chloroform in the presence of one equivalent of pyridine,^104^ yielding the difluoroderivative 151 in 68% yield (without pyridine, the yield was only 40%) (Scheme 39).

The substantial drop in yield in the absence of pyridine (40%) highlights the importance of additive control in optimizing reaction efficiency. From a reactivity standpoint, pyridine plays a crucial role by scavenging HF generated *in situ* and stabilizing reactive sulfur intermediates, thereby suppressing side reactions such as elimination or rearrangement. This additive effect is particularly important in rigid terpenoid frameworks, where neighboring functional groups and steric congestion might otherwise divert the reaction pathway. The same procedure was applied to convert hydroxyketone 152 to 2,2-difluoroallobetulone 153.^24^

In 2002, Slavikova *et al.* reacted hydroxy ketones 154 with DAST to form a single difluoroketone product 155. Optimization of the reaction conditions afforded 38% yield. Enol ketone 156 and alcohol 158 were also used as substrates, providing the desired fluoro derivatives 157 and 159 in 77–80% yield.^105^

Utilization of DAST for converting ketones and aldehydes into geminal difluoro derivatives requires more forcing conditions than alcohol substitution, often resulting in lower yields. For example, aldehyde 160 gave difluoro derivative 161 in only 22% yield after 48 hours, while heptanorketone 162 yielded difluoro derivative 163 in 30%.

**Scheme 40:**
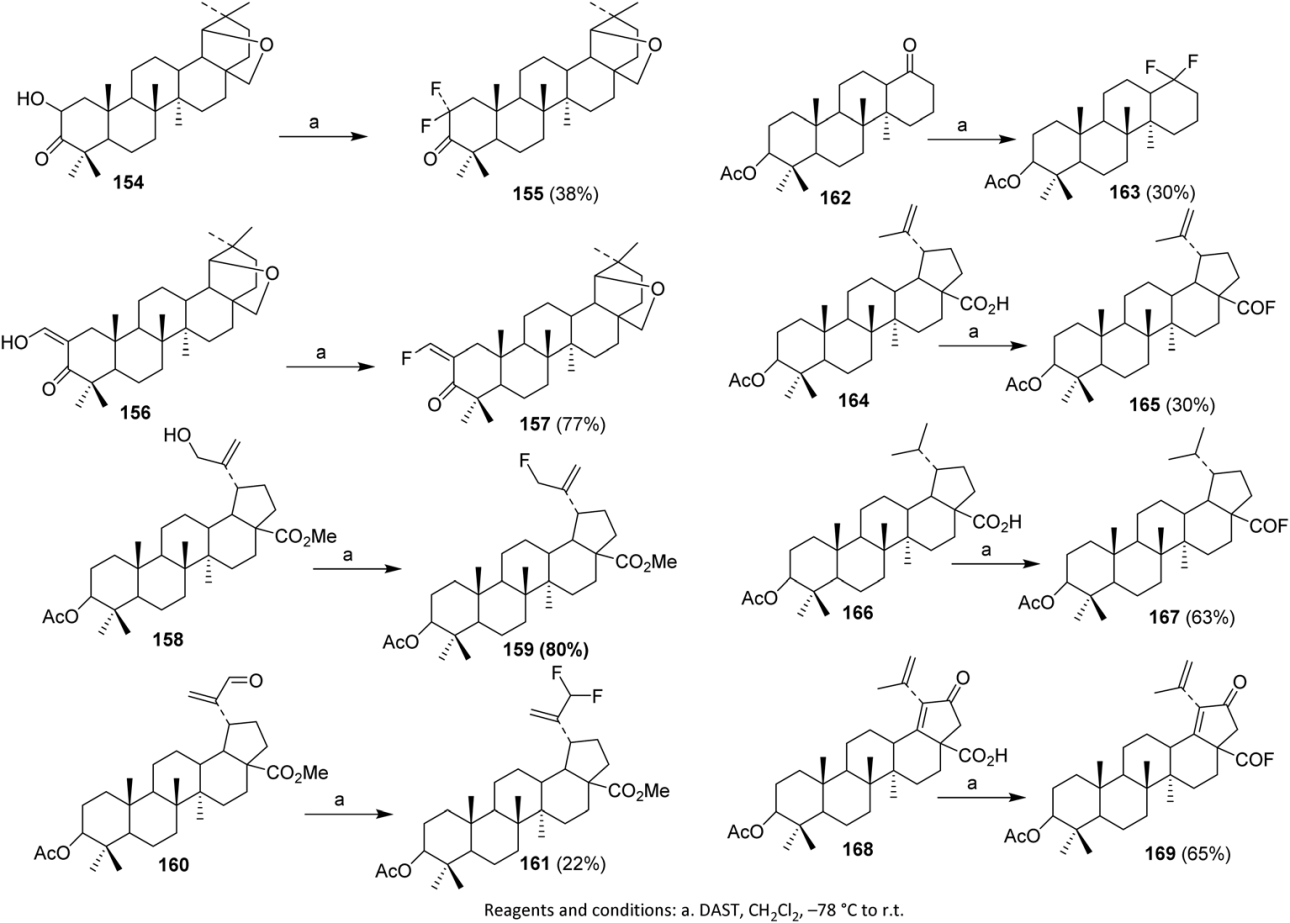
Fluorination of triterpenoids *via* deoxofluorination reaction.^104,105^

DAST is also effective for the synthesis of acyl fluorides under mild conditions. Acetylpreculinic acid 164 was fluorinated to acyl fluoride 165. Biederman *et al.* successfully applied the same procedure to saturated derivatives 166 and 21-oxo acid 168, obtaining the respective acyl fluorides 167 and 169 (Scheme 40).^104^

#### 2.4.3. Amine acylation reaction (*N*-acylation)

**Scheme 41:**
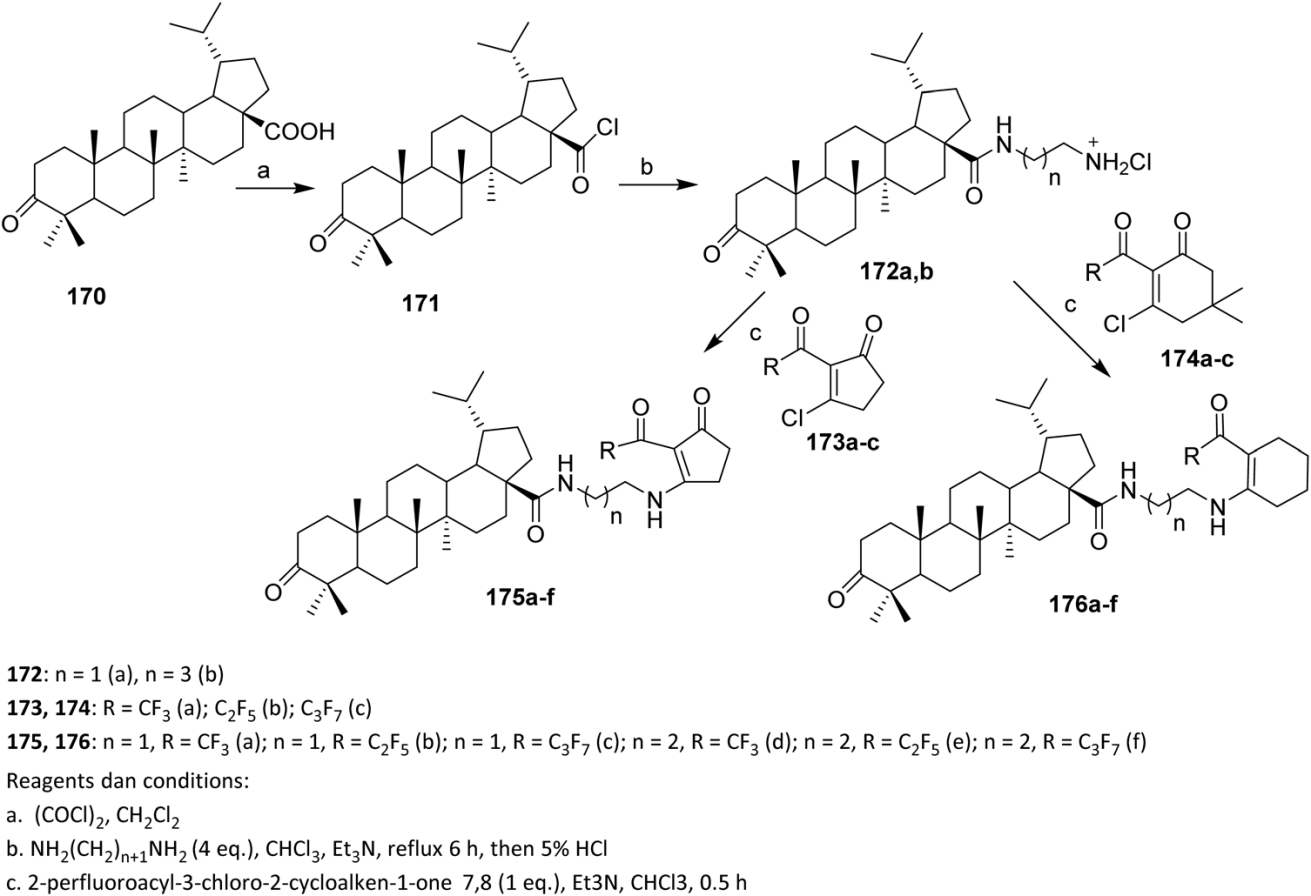
Synthesis of fluorinated triterpenoids *via* amine acylation reaction.^106^

In 2017, Khlebnikova *et al.* conducted a study using pharmacophore conjugation of 2-perfluoroacylcycloalkane-1,3-dione *via* a polymethylenediamine linkage with betulonic acid to produce novel fluorinated biologically active compounds. Betulonic acid 170 was treated with an excess of (COCl)_2_ to synthesize its chloride 171, which was reacted with ethylenediamine or 1,4-butanediamine (the natural polyamine putrescine). The target aminoalkyl hydrochlorides of betulonic acid amide 172a and 172b were obtained in 84% yield after treatment of acid chloride 171 with a four-fold excess of diamine in the presence of Et_3_N in CHCl_3_, followed by work-up with 5% HCl solution. Use of enol derivatives (chlorovinyldiketones, enol esters) in reactions with *N*-nucleophiles could produce endocyclic compounds and regioisomeric exocyclic compounds starting from the triketones. Reaction of 2-perfluoroacyl-3-chloro-2-cycloalken-1-ones 173a–c and 174a–c, with an equivalent amount of betulonic acid amide aminoalkyl hydrochlorides (172a and 172b) in the presence of Et_3_N in CHCl_3_ gave conjugates 175a–f and 176a–f in yields of 84–88% (Scheme 41).^106^

**Scheme 42:**
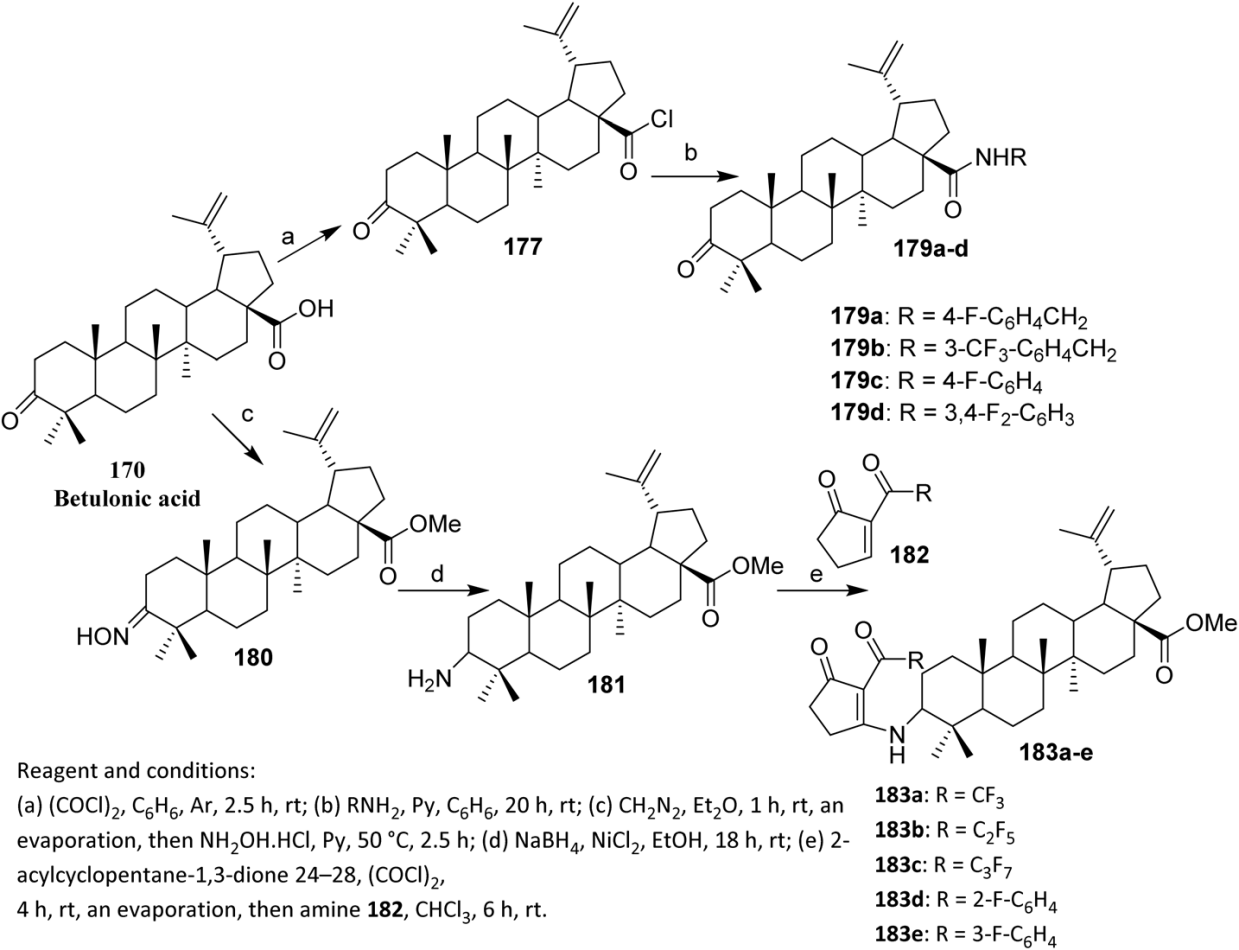
Synthesis of fluorinated amide derivatives of betulinic acid.^107^

The efforts to obtain new anti-inflammatory agents were focused on structural modifications at C-3 and C-28 positions of betulonic acid 170. Scheme 42 shows the methodology used to produce the investigated fluorine-containing amides 179a–d and hybrids 183a–e. To synthesize the fluorinated amide derivatives of betulonic acid, an acylation of the corresponding fluoroaromatic amines with betulonic acid chloride 177, obtained as per literature procedure, was carried out. Compound 177 was allowed to react with the fluorine-containing aromatic amines in dry benzene in the presence of pyridine to produce betulonic acid amides 179a–d in 66–72% yields.

For obtaining the hybrids 183a–e, a reaction of a vinylogous substitution of cyclic *β*-triketone enol derivatives by *N*-nucleophiles was used. Betulonic acid 170 was methylated with diazomethane to give the methyl ester 180. Then oxime 180 was prepared by treatment of this intermediate with hydroxylamine hydrochloride in pyridine. Obtained oxime 180 was reduced under an action of NaBH_4_ in the presence of NiCl_2_ in dry ethanol to give methyl 3-amino-3-deoxybetulinate 181 as a mixture of 3*β*- and 3*α*-isomers (65 : 35 according to ^1^H NMR data). A vinylogous substitution of 2-acyl-3-chlorocyclopent-2-en-1-ones, obtained from corresponding cyclic *β*-triketones, with amine 182 was carried out in chloroform at room temperature to give hybrids 183a–e.^107^

**Scheme 43:**
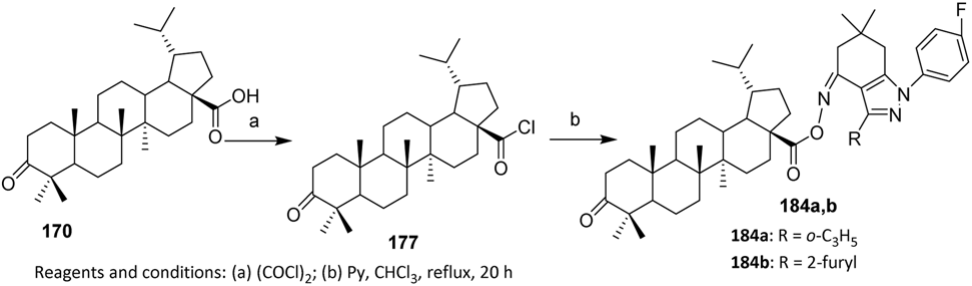
Synthesis of fluorinated heterocyclic derivatives of betulinic acid.^108^

The betulonic acid 170 was transformed into the corresponding chloride 177 according to the previously reported procedure and immediately coupled to the selected oxime. With both key components in hand, a one-pot procedure led to the target conjugates 184a–b. In a typical experiment, a mixture consisting of a selected 6,7-dihydro-1*H*-indazol-4(5*H*)-one oxime, betulonic acid chloride 177, and pyridine in CHCl_3_ was refluxed for 5 h. The acylated products 184a–b were obtained in moderate to good yields (Scheme 43).^108^ From a reactivity and mechanism perspective, the success of the one-pot protocol relies on the high electrophilicity of the acid chloride and the sufficient nucleophilicity of the oxime oxygen. Pyridine plays a dual role as an HCl scavenger and as a mild base, thereby suppressing acid-promoted decomposition of the oxime and minimizing side reactions such as hydrolysis or rearrangement under reflux conditions.

#### 2.4.4. Nucleophilic trifluoromethylation

**Scheme 44:**
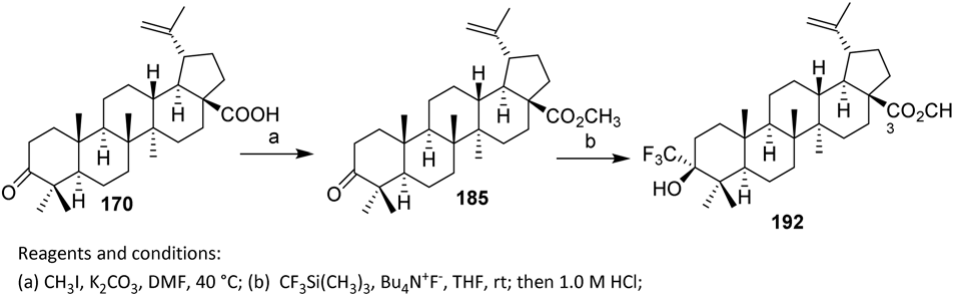
Trifluoromethylation of a betulinic acid derivative using (CF_3_)_3_Si and TBAF.^99,100^

The stepwise strategy reported by Li *et al.* demonstrates a highly site-selective modification of betulonic acid 170, enabling controlled installation of fluorinated functionality without disrupting the rigid pentacyclic framework. In the initial step (a), esterification to afford methyl ester 185 masks the carboxylic acid, thereby preventing acid–base interference in subsequent transformations and preorganizing the molecule for selective functionalization at the carbonyl-bearing C-3 position (Scheme 44).

In step (b), 185 is treated with CF_3_Si(CH_3_)_3_ (Ruppert–Prakash reagent) and Bu_4_N^+^F^−^ in THF at room temperature, followed by acidification with HCl, to introduce a trifluoromethyl group at the C-3 position. This nucleophilic trifluoromethylation produces the final compound 192, functionalized with both a methyl ester at C-28 and a CF_3_ substituent at C-3, enhancing its chemical and biological properties. The mild reaction conditions and controlled acidic quench favor clean trifluoromethyl addition over competing reduction or elimination pathways, underscoring effective chemoselectivity in a densely functionalized triterpenoid scaffold.

In terms of substrate scope, this methodology is best suited to ketone-containing triterpenoids analogous to betulonic acid, particularly those featuring an accessible and relatively unhindered carbonyl group. The approach therefore suggests broader applicability to other lupane- or oleanane-type scaffolds, provided that the carbonyl functionality is sufficiently reactive toward nucleophilic trifluoromethylation.^99,100^

Piperidine derivative 193 was obtained by replacement of the terminal chloride with an amine, in which the amine acts as a strong nucleophile to displace the chloride under controlled conditions. This transformation demonstrates high chemoselectivity, as substitution occurs preferentially at the activated terminal position without affecting other functional groups within the molecule.

**Scheme 45:**
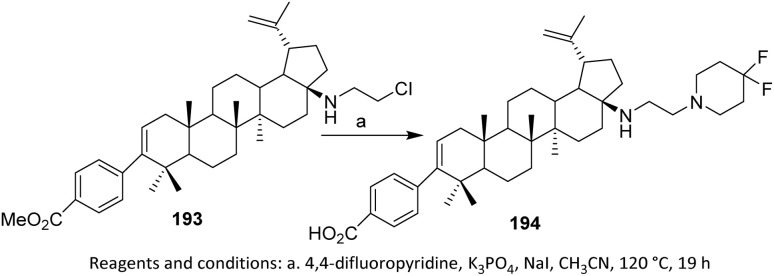
Formation of a 4,4-difluoropiperidine-linked steroidal derivative.^109^

Subsequent deprotection of the benzoate ester furnishes compound 194, restoring the free hydroxyl group and increasing the polarity of the final product. The stepwise sequence allows independent control over C–N bond formation and protecting-group removal, which is advantageous when working with multifunctional terpenoid or alkyl halide frameworks (Scheme 45).^109^

#### 2.4.5. Esterification

**Scheme 46:**
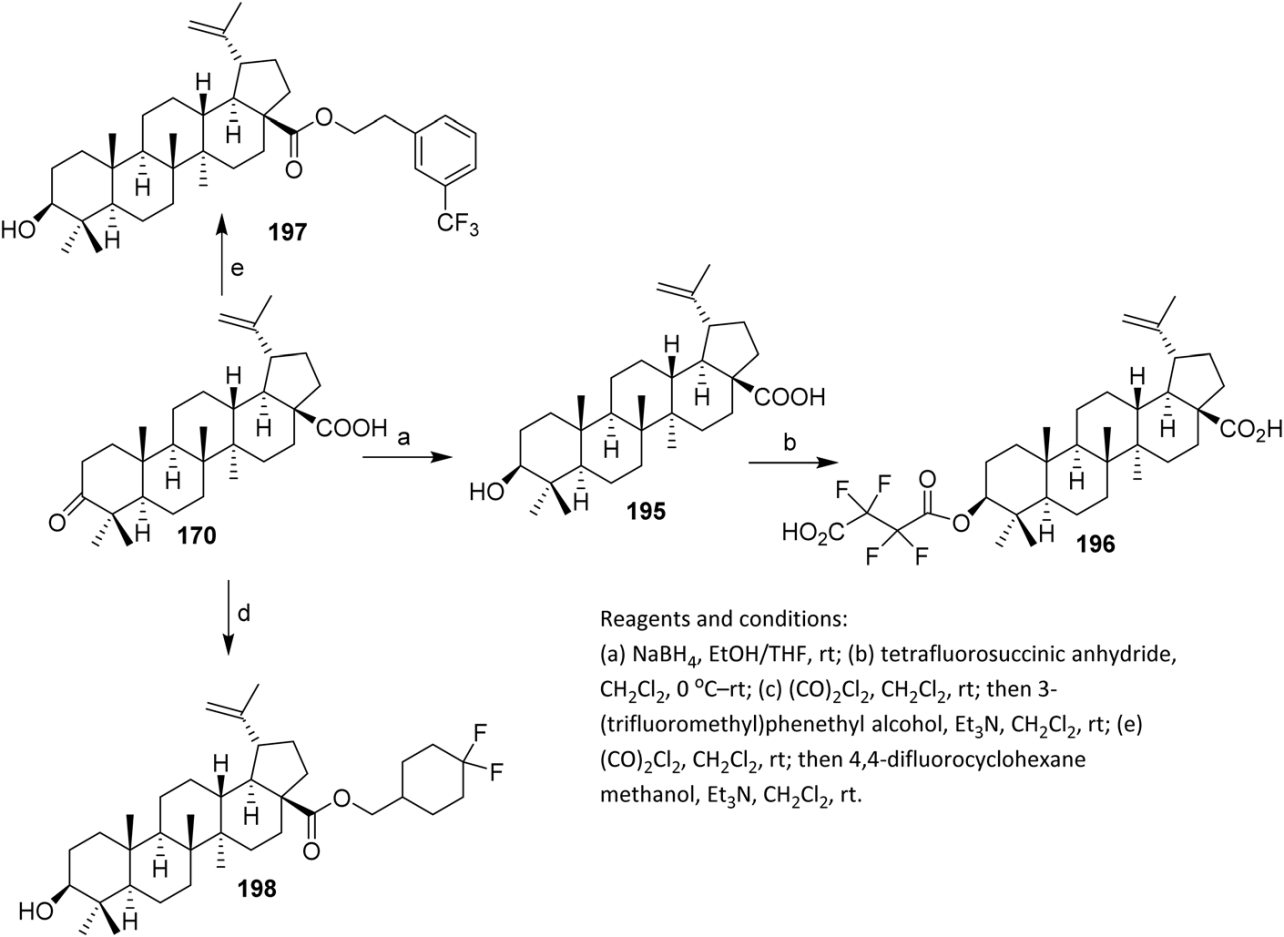
Synthesis of fluorinated ester and ether derivatives of betulinic acid.^99,100^

In addition to electrophilic and nucleophilic approaches, in 2017, Li *et al.*^99^ also employed esterification as an effective method for fluorination of the scaffold. In Scheme 46, a series of modifications on 170 are illustrated to generate fluorinated derivatives. Initially, 170 was reduced with NaBH_4_ in EtOH/THF to afford 195. This was followed by esterification with tetrafluorosuccinic anhydride in CH_2_Cl_2_, producing intermediate 196. Further derivatization of 170 was achieved through esterification with 3-(trifluoromethyl)phenethyl alcohol in the presence of Et_3_N, giving 197 with a trifluoromethyl-substituted side chain. In a parallel pathway, 198 was synthesized *via* reaction with (COCl)_2_ in CH_2_Cl_2_ and subsequent treatment with 4,4-difluorocyclohexane methanol. Overall, these transformations highlight several fluorination strategies, such as esterification with fluorinated anhydrides and alcohols, to diversify the betulinic acid framework for potential biological applications.^99,100^ However, the limitations of this approach stem from the indirect nature of fluorine incorporation, as fluorine is introduced exclusively as part of peripheral substituent fragments rather than being directly installed onto the triterpenoid skeleton. Consequently, this strategy exerts a less pronounced influence on the intrinsic electronic properties of the core, and its biological impact is highly dependent on ester stability and susceptibility to hydrolysis under physiological conditions.

#### 2.4.6. Sonogashira cross-coupling reaction (Pd/Cu-catalyzed alkynylation)

**Scheme 47:**
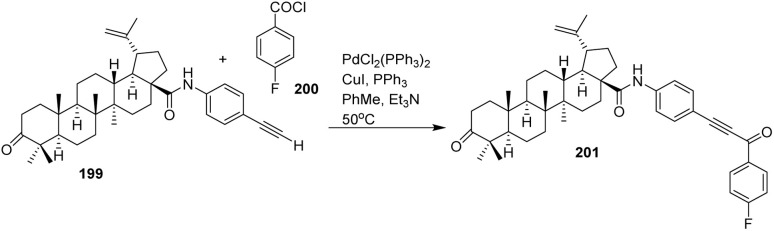
Fluorination of a betulinic acid derivative *via* Sonogashira coupling reaction.^3^

In 2022, Semenova *et al.* reported the modification of a betulinic acid (BA) derivative *via* a Sonogashira coupling reaction, enabling the incorporation of a fluoroarene-containing substituent (200), as illustrated in Scheme 47. The Sonogashira coupling represents a highly chemoselective C–C bond-forming strategy for incorporating a fluorinated aromatic fragment into the betulinic acid (BA) framework. The use of a terminal alkyne–functionalized BA derivative 199 confines reactivity to the alkyne site, enabling selective attachment of the fluorinated aryl unit without perturbing the densely functionalized triterpenoid core.

From a reactivity and catalytic standpoint, the PdCl_2_(PPh_3_)_2_/CuI system efficiently mediates oxidative addition of the aryl chloride 200, transmetalation with the copper acetylide, and subsequent reductive elimination to form the Csp–Csp^2^ bond. Triethylamine serves both as a base and as a scavenger for HX, while toluene at moderate temperature (50 °C) balances catalytic activity with suppression of alkyne homocoupling or decomposition. The catalytic system enables the formation of a new Csp–Csp^2^ bond between the alkyne and aryl chloride, leading to the integration of a fluorinated aromatic group onto the BA structure and producing 201.

In terms of substrate scope, this approach is well suited for BA derivatives bearing terminal alkynes and for aryl halides containing fluorinated substituents, allowing modular introduction of diverse fluorinated aromatic motifs. The strategy is potentially extensible to other triterpenoid scaffolds, provided that the alkyne functionality is sterically accessible and compatible with palladium catalysis.^3^

#### 2.4.7. Oxidation–reduction reaction

**Scheme 48:**
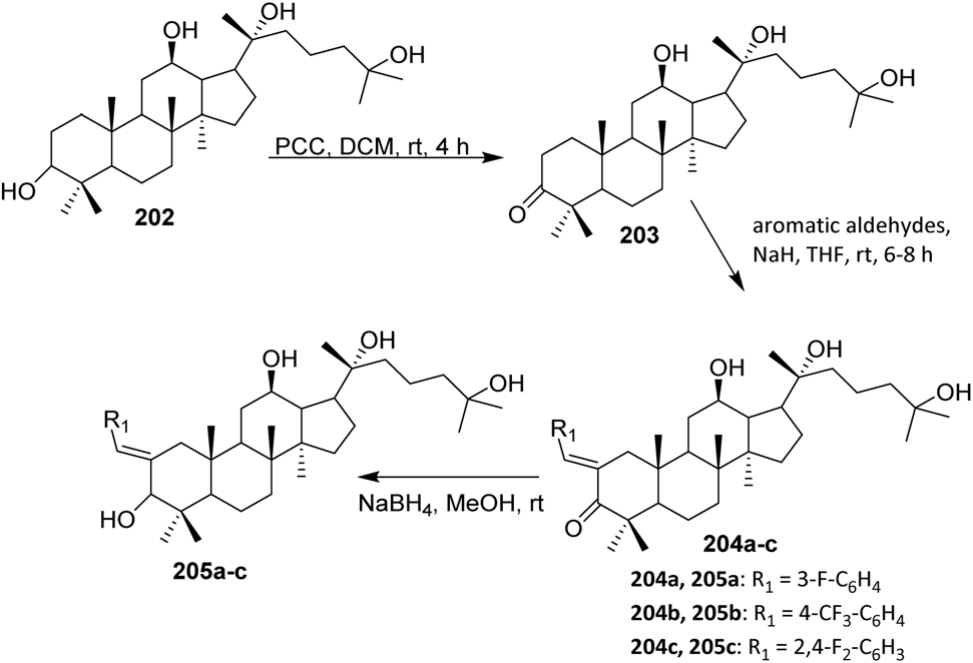
Fluorination of a triterpenoid *via* an oxidation–reduction reaction.^2^

The synthetic sequence in Scheme 48 highlights a chemoselective and modular approach to diversify the C-3 position of scaffold 202 through oxidation–condensation–reduction steps. Oxidation with pyridinium chlorochromate (PCC) cleanly converts the C-3 hydroxyl into ketone 203, providing a well-defined electrophilic center for subsequent aldol condensation with aromatic aldehydes, which proceeds selectively to give benzylidene derivatives 204a–c without affecting other functional groups.

From a reactivity control perspective, the use of sodium hydride (NaH) in tetrahydrofuran (THF) promotes efficient enolate formation at C-3, enabling controlled C–C bond formation with a range of aromatic aldehydes. The subsequent sodium borohydride (NaBH_4_) reduction selectively reduces the C-3 carbonyl in 204a–c to regenerate the hydroxyl group in 205a–c, while preserving the newly installed benzylidene moiety, demonstrating good functional group tolerance and predictable chemoselectivity.^2^

#### 2.4.8. Ozonolysis of an alkene

**Scheme 49:**
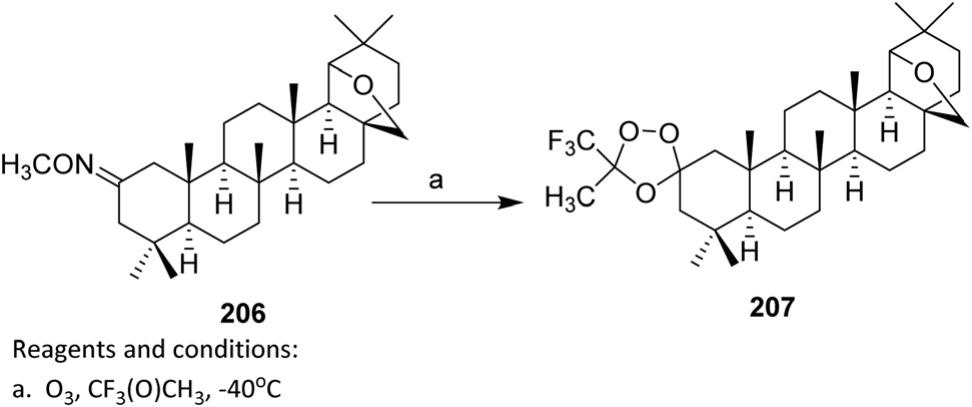
Oxidative ozonolysis of a steroidal alkene to a trifluoroacetate derivative.^110,111^

The ozonolysis of isomeric mixtures of compounds 206 constitutes a highly efficient and chemoselective route to nonsymmetrical spiro-1,2,4-trioxolanes 207*via in situ* generation of carbonyl oxide intermediates. Performing the reaction at low temperature (−40 °C) in a cyclohexane–CH_2_Cl_2_ solvent system minimizes overoxidation and decomposition, enabling controlled interception of the reactive dipoles and affording the desired ozonides in yields of up to 81% (Scheme 49).^110^

From a reactivity and mechanistic perspective, the use of fluorinated ketones such as CF_3_C(O)CH_3_ is crucial. The strongly electron-withdrawing CF_3_ group markedly enhances the dipolarophilicity of the carbonyl moiety, thereby promoting rapid and selective [3 + 2] cycloaddition with the carbonyl oxide intermediate. This electronic activation offsets steric constraints imposed by the bulky terpenoid framework and directs efficient spirocyclization.

With respect to substrate scope, the successful conversion of isomeric mixtures of 205 indicates a notable tolerance toward olefin geometry and substitution patterns. Consequently, this strategy is particularly attractive for late-stage diversification of complex terpenoid scaffolds, especially when fluorinated dipolarophiles are employed to drive cycloaddition efficiency.^111^

#### 2.4.9. Comparative summary of fluorination strategies for triterpenoids

Fluorination methodologies applied to triterpenoids display a broad spectrum of reactivity, encompassing direct C–F bond formation, functional group interconversion, and late-stage scaffold diversification. Electrophilic fluorination using reagents such as Selectfluor, NFSI, CF_3_OF, and NOF has proven particularly effective for modifying activated sites, including enones, enolates, and lactone-forming motifs, in pentacyclic frameworks such as asiatic acid, ursolic acid, and betulinic acid derivatives. These transformations often exhibit pronounced regio- and stereoselectivity governed by substrate control, exploiting the rigid triterpenoid architecture and conjugation effects. Nevertheless, their broader applicability is limited by harsh reaction conditions (*e.g.*, elevated temperatures and strongly electrophilic reagents), safety concerns associated with highly reactive fluorinating agents (notably CF_3_OF and NOF), and a narrow substrate scope, as unactivated C–H bonds or protected carboxylic acids are generally unreactive.

Deoxofluorination and nucleophilic fluorination approaches provide complementary routes to mono- and *gem*-difluorinated triterpenoids, enabling substantial electronic perturbation of the core scaffold. DAST-mediated deoxofluorination allows late-stage substitution of hydroxyl or carbonyl functionalities, even in structurally complex systems; however, yields are strongly dependent on steric effects, reaction conditions, and the nature of the functional group (alcohols *versus* carbonyls). Nucleophilic trifluoromethylation using the Ruppert–Prakash reagent enables site-selective installation of CF_3_ groups at carbonyl positions under relatively mild conditions, offering high chemoselectivity and expanded physicochemical diversity. Despite these advantages, such methods are largely confined to substrates bearing reactive carbonyl groups and may suffer from reduced efficiency or competing side reactions in more elaborate molecular settings.

Indirect fluorination strategies—including esterification, amide formation, cross-coupling reactions (*e.g.*, Sonogashira coupling), oxidation–reduction sequences, and ozonolysis—constitute some of the most versatile and reliable approaches for generating libraries of fluorinated triterpenoids. These methods generally exhibit excellent functional group tolerance, predictable regioselectivity, and compatibility with late-stage diversification, enabling the modular incorporation of fluorinated aromatic, heterocyclic, or aliphatic fragments. Their principal limitation lies in the fact that fluorine is introduced as a peripheral substituent rather than being directly embedded within the triterpenoid carbon framework, thereby exerting a more modest influence on the intrinsic electronic properties of the core. Consequently, no single fluorination strategy is universally optimal for triterpenoids; instead, the selection of an appropriate method depends on the targeted position, the desired fluorine motif (F, CF_2_, or CF_3_), scaffold sensitivity, and the balance between synthetic efficiency and structural impact.

### 2.5. Fluorinated tetranorterpenoids (limonoids)

Limonoids, a group of highly oxygenated triterpenoids, mainly exist in the Rutaceae and Meliaceae plant families. Tetranortriterpenoids is an alternative name for limonoids because, during oxidative changes of triterpenoids, the side chain is eventually oxidized to an α-substituted furyl ring by the loss of four carbon atoms. Limonoids exhibit a wide spectrum of biological properties, including cytotoxic, antioxidant, anti-inflammatory, neuroprotective, antiviral, antimicrobial, antiprotozoal, antimalarial, insect antifeedant, and insecticidal activities.^112^

#### 2.5.1. Deoxofluorination Reaction

**Scheme 50:**
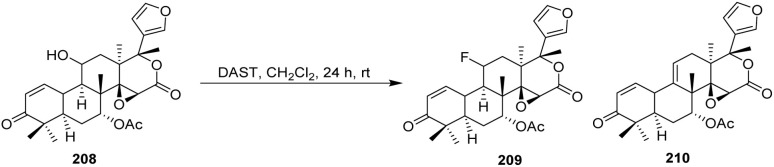
Fluorination of limonoid using DAST.^114^

DAST is frequently used for the preparation of fluorinated compounds, converting alcohols into fluorides under very mild conditions. The DAST-mediated transformation of 11α-hydroxygedunin 208 demonstrates stereoselective deoxofluorination within a densely functionalized limonoid framework. 11α-Hydroxygedunin 208, isolated from *Cedrela sinensis*,^113^ undergoes fluorination predominantly with inversion at C-11 to afford 11β-fluorogedunin 209 in 55% yield (Scheme 50). This outcome is consistent with an SN2-type displacement pathway and highlights the ability of DAST to deliver controlled stereochemical outcomes even in complex natural product scaffolds.^114^

From a reactivity standpoint, the requirement for a large excess of DAST (10 equiv.) and an extended reaction time (24 h) reflects both steric hindrance at the secondary alcohol and the presence of competing reaction pathways. The formation of dehydrated by-product 210 in 10% yield indicates partial elimination, likely promoted by *in situ* – generated HF, underscoring the delicate balance between substitution and elimination in DAST-based transformations.^114^

#### 2.5.2. Reduction of hydroxy limonoid

**Scheme 51:**
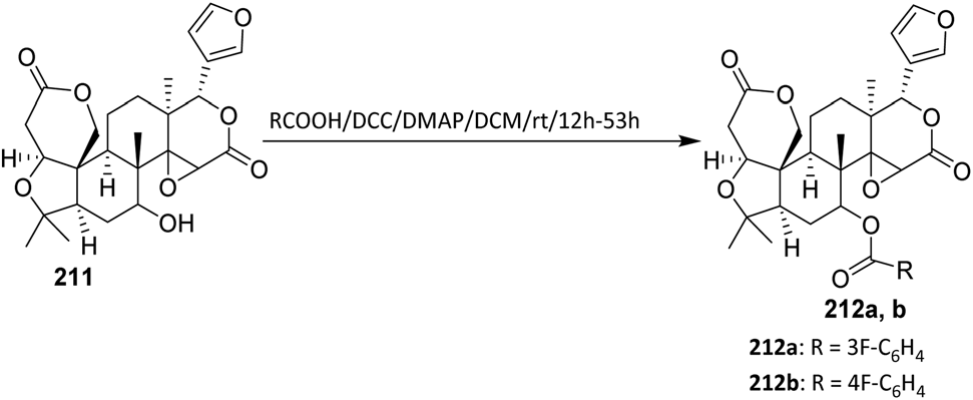
Fluorination of limonoid *via* esterification using DCC/DMAP.^116^

Limonin 210, the most abundant limonoid from *Citrus*, is a typical representative of this class.^115^ The modification of limonin 210*via* DCC/DMAP-mediated coupling represents a chemoselective esterification strategy for derivatizing a complex limonoid scaffold while preserving its highly oxygenated framework. Activation of the carboxylic acid by DCC enables efficient acyl transfer to the accessible hydroxyl group of limonin, affording derivatives 211a and 211b without perturbing the sensitive lactone and furan moieties (Scheme 51). From a reactivity and selectivity standpoint, DMAP plays a crucial catalytic role by accelerating acyl transfer and suppressing side reactions, such as O → N acyl migration or decomposition of activated intermediates. The success of this transformation demonstrates that limonin tolerates mild carbodiimide-mediated conditions despite its considerable structural complexity.^116^

**Scheme 52:**
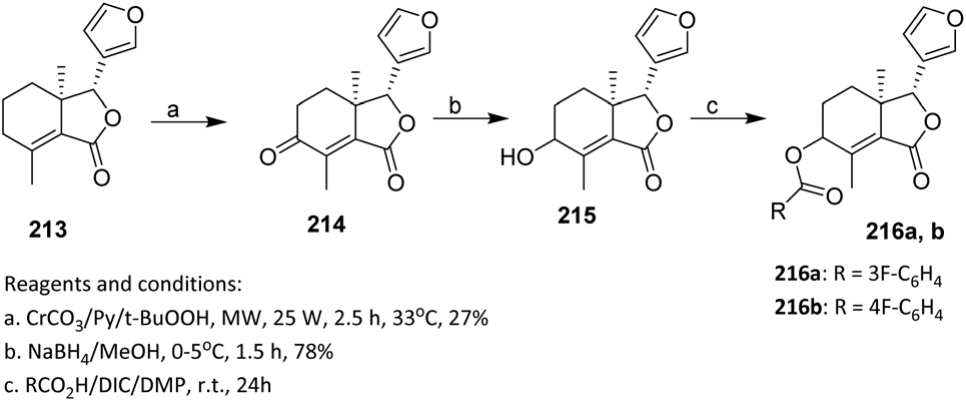
Fluorination of limonoid *via* esterification using DIC/DMAP.^117^

The sequence shown in Scheme 52 illustrates a regioselective, stepwise functionalization strategy for modifying the fraxinellone scaffold. The initial chromium trioxide–mediated allylic oxidation under microwave irradiation selectively targets the allylic position of 213 to afford fraxinellone 214, demonstrating effective site selectivity despite the presence of multiple oxidizable sites within the limonoid framework. From a reactivity control perspective, microwave irradiation accelerates the oxidation process and enhances regioselectivity by promoting rapid and uniform heating. Subsequent NaBH_4_ reduction cleanly converts the carbonyl group of 214 into alcohol 215 without affecting other sensitive functionalities. This reduction step reintroduces a reactive hydroxyl handle, thereby enabling further derivatization.

The substrate scope of the final esterification step is governed by the accessibility of the hydroxyl group in 215 and the nature of the carboxylic acids employed. The use of DIC/DMAP enables efficient coupling with different acids to afford esters 216a and 216b, indicating reasonable tolerance toward varied acyl substituents and suitability for structure–activity relationship (SAR)-driven diversification.

However, this approach is limited by the relatively low yield of the initial oxidation step (27%) and the reliance on chromium-based oxidants, which raise concerns regarding environmental impact and functional group compatibility.^117^

#### 2.5.3. Fluorination *via* borylation and Selectfluor

**Scheme 53:**
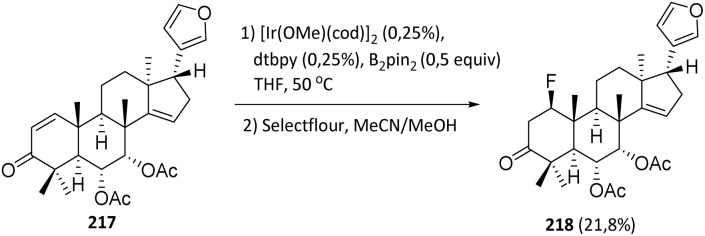
One-pot, two-step borylation/fluorination reaction of dysobinin.^118^

Dysobinin 217, the major limonoid isolated from *C. macrophyllus*, is considered a representative compound of the limonoid class. Huda and Budiman *et al.* conducted its fluorination using a two-step, one-pot method: first, an Ir-catalyzed C–H borylation, followed by fluorination with Selectfluor. This approach addresses the instability of the heteroaryl pinacol boronate intermediate, which, if exposed to oxygen, can revert to the starting dysobinin 217. The reaction was therefore performed under inert atmospheric conditions. The resulting 1*β*-fluorodysobinin 218 was obtained as white crystals with molecular formula C_30_H_39_O_6_F, confirmed by HR-TOF-MS (*m*/*z* 515.2808 [M + H]^+^, calcd 515.2809), indicating twelve degrees of unsaturation. Compound 218 exhibited significantly higher cytotoxicity against A549 lung cancer cells compared to dysobinin (Scheme 53).^118^

#### 2.5.4. Comparative summary of fluorination strategies for tetranorterpenoids

Fluorination methodologies for limonoids, a highly oxygenated class of tetranortriterpenoids, illustrate the balance between direct C–F bond formation and indirect functionalization strategies. DAST-mediated deoxofluorination enables stereoselective substitution of secondary alcohols within rigid limonoid frameworks, as demonstrated by the conversion of 11α-hydroxygedunin to 11β-fluorogedunin *via* an SN2-type mechanism. While this approach affords controlled stereochemical outcomes, it is limited by steric hindrance, the requirement for excess reagent, extended reaction times, and competing elimination pathways. In contrast, indirect fluorination through esterification (DCC/DMAP or DIC/DMAP) offers a milder and more versatile strategy, enabling late-stage derivatization of limonin and fraxinellone while preserving sensitive functional groups. This method is well suited for structure–activity relationship (SAR) studies, as fluorine is introduced exclusively as a peripheral substituent. More advanced borylation–Selectfluor sequences allow direct, site-selective C–H fluorination of the limonoid core, exemplified by fluorinated dysobinin with enhanced cytotoxicity; however, broader application remains constrained by catalyst cost, reaction sensitivity, and narrow substrate scope.

## 3. Conclusion

Fluorinated terpenoids exhibit diverse and significant bioactivities, underscoring their growing importance in medicinal and synthetic chemistry. In the past, the fluorination of terpenoids often resulted in product mixtures with low conversions and poor overall yields. Moreover, the use of fluorinating reagents required extensive precautions during synthesis, and some reagents such as perchloryl fluoride are considered highly hazardous. These factors have limited the practical utilization of fluoro-substituted terpenoids.

However, the development and commercialization of new electrophilic and nucleophilic fluorinating agents, along with the emergence of metal-catalyzed fluorination reactions, have eliminated the need for direct handling of elemental fluorine in pharmaceutical laboratories. These advances have opened efficient pathways for the selective introduction of fluorine atoms into terpenoid frameworks with high yields. Among the most widely used reagents are DAST, trifluoromethyl hypofluorite, Selectfluor, and NFSI, which are readily available for electrophilic fluorination. Meanwhile, nucleophilic fluorination agents are tetrabutylammonium fluoride hydrate (TBAF*·*3H_2_O), triethyl(trifluoromethyl)silane (TESCF_3_), and Ruppert—Prakash reagent (TMSCF3—TBAT/TMAF). In addition, fluorination can also be achieved through the incorporation of aryl groups containing fluorine atoms. This approach encompasses various reaction types, including esterification, alcohol reduction, michael addition, oxidation–reduction, C–C cross-coupling (*e.g.*, Heck coupling and Sonogashira coupling), click chemistry, ozonolysis of an alkene and *N*-acylation reactions (Table 1).

**Table 1:** Summary of fluorination strategies across terpenoid classes

Strategy type	Monoterpenoids	Sesquiterpenoids	Diterpenoids	Triterpenoids	Tetranortriterpenoids
Functional group transformation–driven approaches	Esterification; Prins-type reactions; acid-catalyzed transformations	Michael addition; acylation reactions; functional group interconversions	Esterification; oxidation–reduction; ozonolysis of alkenes	Esterification and multistep functional modification	Reduction of hydroxy limonoids; functional group manipulation
Nucleophilic fluorination/deoxofluorination	Alcohol-based deoxofluorination; site-selective C–F installation	Limited application due to skeletal complexity	Deoxofluorination reactions; nucleophilic trifluoromethylation	Applicable but requires pre-functionalized substrates	Deoxofluorination of hydroxylated limonoids; late-stage modification
Electrophilic fluorination	Applicable but limited regioselectivity	Electrophilic fluorination of activated sites	Direct electrophilic fluorination with good functional-group tolerance	Sterically hindered; limited direct application	Electrophilic fluorination following borylation
Metal-catalyzed & cross-coupling strategies	Pd- and Cu-catalyzed transformations; click reactions	Pd-catalyzed cross-coupling reactions	Pd/Cu-catalyzed cross-coupling; alkynylation	Limited due to steric congestion	Borylation–fluorination sequences enabling scaffold diversification
Overall synthetic features	Simple skeletons favor acid-catalyzed and functional-group-based strategies	Moderate complexity allows multiple fluorination pathways	Larger frameworks tolerate diverse and selective fluorination methods	Structural rigidity limits direct fluorination	Highly functionalized scaffolds favor controlled, multistep strategies

Despite these advances, several challenges remain and will likely define future research directions in this field. Achieving site-selective and stereoselective fluorination on complex terpenoid scaffolds continues to be difficult, particularly for late-stage functionalization. Furthermore, many reported transformations rely on stoichiometric reagents or multistep sequences, highlighting the need for more sustainable, catalytic, and environmentally benign fluorination strategies. From a biological perspective, systematic structure–activity relationship studies are still limited, and deeper insight into how fluorine incorporation influences target selectivity, metabolic stability, and toxicity is required. Addressing these challenges will be essential for translating fluorinated terpenoids from synthetic curiosities into practical candidates for medicinal, agrochemical, and materials applications.

## Conflicts of interest

There are no conflicts to declare.

## Data Availability

No new primary research data, software, or code were generated or analysed in the preparation of this review.
